# Generative AI, Health Misinformation, and Medical Deepfakes: Implications for Diagnostic Safety and Clinical Practice—A Narrative Review

**DOI:** 10.3390/diagnostics16213572

**Published:** 2026-10-29

**Authors:** İsmail Baydili, Burak Tasci, Sengul Dogan, Turker Tuncer

**Affiliations:** 1 Department of Audiovisual Techniques and Media Production, Vocational School of Technical Sciences, Fırat University, Elazig 23119, Türkiye; 2 Vocational School of Technical Sciences, Fırat University, Elazig 23119, Türkiye; 3 Department of Digital Forensics Engineering, Technology Faculty, Fırat University, Elazig 23119, Türkiye; sdogan@firat.edu.tr (S.D.); turkertuncer@firat.edu.tr (T.T.)

**Keywords:** generative artificial intelligence, health misinformation, medical deepfakes, diagnostic safety, clinical decision support, trustworthy AI

## Abstract

Generative artificial intelligence (AI) can rapidly produce persuasive health information in the form of text, synthetic images, cloned voices and deepfake videos. It can also be used to create fictitious professional identities. This narrative review describes how synthetic claims, fabricated evidence and simulated identities are used to disguise misleading health information and give it the appearance of authenticity especially with regard to diagnostic safety and clinical practice. Literature identified through Web of Science and Scopus was synthesized across text image audio video and multimodal formats; current evidence indicates that generative AI can produce highly credible but inaccurate health content. Source authority, message framing and professional identity cues influence trust and verification behavior, while disease-specific evaluations show that high average accuracy may coexist with clinically important omissions, missed referrals, hallucinated findings and poor calibration. In medical imaging, errors may arise both from incorrect interpretation and from manipulation of the diagnostic evidence itself. Detection, evidence-based verification, provenance, literacy interventions, and governance provide complementary safeguards, although real-world and longitudinal evidence remains limited. Clinical evaluation should therefore consider task-specific performance, source authenticity, human oversight, and the consequences of individual errors alongside overall accuracy.

## 1. Introduction

Social media provides a major setting for creating, sharing, and discussing health information. Health professionals, patients, content creators, and commercial actors circulate diverse information and experiences on the same platforms. While this environment facilitates access to reliable health information, it also allows claims without scientific basis to gain visibility. Research on health misinformation on social media points to a problem area that varies depending on the platform and the content examined, covering topics such as vaccines, diseases, treatments, and health products [1].

Generative AI enables the rapid generation of different types of false health information and can be tailored to specific user groups. Menz et al. demonstrated the generation of health disinformation materials such as fabricated patient- and clinician-centered narratives as well as sources with a scientific appearance using large language models and visual generation tools [2]. However, generating content is not the same as studying its communicative impact. An experiment by Spitale et al. used tweet-formatted health messages and found that in the conditions of the experiment participants were unable to determine whether AI-generated or human-generated disinformation had been communicated. Furthermore, the AI-generated disinformation was found to be more persuasive than human-generated disinformation [3]. Therefore, distinguishing between accurate content and health misinformation, a source’s identity, and information’s perceived credibility must all be separately evaluated.

In health communication, the identity of the person presenting a claim adds a further dimension to its perceived credibility. Xie and Li [4]. analyzed health news generated by AI and found that the source of information, whether the message is presented in a positive or negative emotional manner, and the communication channel all impact the ability to identify misinformation. An opinion piece on “doctor deepfakes” describes how the identities of doctors are currently being misused in order to spread misinformation [5]. Thus, our scope of analysis goes from a single false health claim to the claim’s author and the evidence that supports it. Note that accuracy assessment and AI detection are two different tasks, and that is also reflected by the MM-Health dataset that contains health content with text and images [6]. Misleading health information spread online can affect decisions about disease prevention and treatment. The effect of misleading online health information has been studied, including a randomized study published by Loomba et al. which showed that exposure to online misinformation about COVID-19 vaccines could decrease the vaccination intent [7]. However, this finding does not establish whether AI-generated misinformation, including deepfakes, has effects of the same magnitude. The key is to distinguish between information being spread online, believed by readers, shared by readers, health prevention and treatment intent by readers, health information-related behavior by readers, clinical harm, and more. Saeidnia et al. [8] reviewed the production and dissemination of AI-generated health misinformation and potential countermeasures.

The review is discussed from a diagnostic safety perspective and clinical impact and explores the interrelation of health misinformation that is generated synthetically, fabricated evidence of clinical trials, health misinformation disseminated under the impersonated professional identities of doctors and health experts and fabricated artificial authority of health professionals. Three inter-related risk pathways have been identified in the review including intentionally created health misinformation by AI, unintended errors by AI models that are used by patients and healthcare professionals for decision-making and diagnostic evidence that has been created synthetically for diagnostic purposes.

Five research questions guide this review of health misinformation, disinformation, and deepfakes across text, image, audio, and video formats:

- **RQ1:** How does generative AI change the ways in which misleading health claims, supporting evidence, and source identities are produced for social media?
- **RQ2:** How do source identity, expertise cues, message format, and platform context affect credibility, persuasion, verification, and sharing?
- **RQ3:** What evidence supports the relationship between exposure to this content and the use of generative AI and health-related beliefs, diagnostic decisions, behaviors, and clinical harm?
- **RQ4:** What are the effectiveness and limitations of detection, verification, communication interventions, and governance approaches?
- **RQ5:** Through which error pathways can generative AI systems and synthetic medical content affect diagnostic safety, and what criteria should be used in the clinical validation of these risks?

## 2. Method

### 2.1. Design of the Review

This study was designed as a narrative review addressing AI-generated health misinformation, disinformation, and deepfake content in the context of social media and health communication. This review examines the evolution of AI-generated health misinformation from synthetic claims to fabricated evidence, synthetic patient narratives, and the impersonation of healthcare professionals. The aim is to relate the modes of production of this content to credibility, persuasion, expert authority, and health-related outcomes. In structuring the methodology, SANRA’s criteria regarding the articulation of objectives, the definition of the literature search, appropriate citation, and scientific reasoning were adopted as a guide [9].

### 2.2. Literature Review and Reference Management

The literature review was conducted in the Web of Science Core Collection and Scopus databases using complementary searches tailored to the thematic structure of the review. The initial search was followed by complementary searches focusing on synthetic media and identity impersonation, social media and health communication, as well as detection, intervention, and governance.

Health-related terms were combined with concepts such as generative artificial intelligence, large language models, misinformation, disinformation, hallucinations, deepfakes, and synthetic media. In disease-specific and clinically focused supplementary searches, terms such as diagnostic safety, clinical safety, diagnostic error, patient safety, medical imaging, and synthetic medical images were also used. Depending on the focus of the search, terms related to social media platforms, verification, tagging, misinformation correction, and moderation were added.

For the purpose of the review the literature retrieved was discussed using a narrative approach and included details of the methodology used in individual studies, their clinical relevance and the value of the findings. Studies were selected on the basis of the review questions and included empirical studies, methodological investigations and reviews of a scholarly nature. The evidence was assessed using criteria related to the design of the studies, their clinical setting and the results reported. A distinction was made between findings that had been experimentally proven and their potential for application in clinical settings. As the review was of an exploratory, interdisciplinary nature, the synthesis of the evidence provided was by means of a critical integration of all of the evidence retrieved, as opposed to a complete systematic overview or quantitative meta-analysis.

### 2.3. Scope and Analytical Framework

This review examines how AI is used to create misleading health claims, fabricated evidence, and synthetic identities, alongside their effects on health communication and approaches to mitigation. The origin of content in AI alone does not establish that content is misinformation. The classification of disinformation requires evidence of intentional deceit. Material which is presented as part of an educational simulation, anonymized data, or part of an augmented dataset is not considered to be misinformation simply because the material is synthetic in nature. The material must also be accurate and presented properly in the given context. Studies were examined by content modality (text, image, audio, video, or multimodal), function (production, dissemination, perception, detection, intervention, or governance), misinformation type, health domain, and study design. The social media platform, target audience, impersonated identity, and measured outcomes are dimensions that complement the interpretation of findings in the context of health communication and clinical practice. Disease-specific clinical studies were also evaluated in terms of diagnostic tasks, critical error types, referral safety, clinical validation, and potential patient safety implications. During the analysis, intentional synthetic disinformation, unintentional model error, and synthetic or manipulated diagnostic evidence were treated as distinct risk pathways.

For purposes of this synthesis, production capacity, dissemination, perceived credibility, behavior change, and clinical harm were treated as distinct outcomes. That evidence found at a particular stage in the review did not necessarily mean that same evidence would find expression at subsequent stages. Results related to the technical performance of various AI systems, experimental findings, and conceptual discussions were not treated as equivalent. Gaps identified in the reviewed literature do not necessarily indicate an absence of research on those topics. While the experimental studies reported here established effects under controlled circumstances, the various forms of observational evidence provided primarily associations, and individual case reports described isolated instances. While these various types of evidence pointed out a number of vulnerabilities in health communication and diagnostic practice, there was, however, a significant dearth of evidence establishing a connection between AI-generated misinformation (including medical deepfakes) and sustained changes in behavior as well as clinical detriment.

## 3. Conceptual Boundaries

For generated health content online, separate assessments are required for factual accuracy, generation method, intent to deceive, and online source. The terms health misinformation, health disinformation, health-related AI hallucinations, synthetic health-related online media, and health-related deepfakes all describe somewhat different dimensions and thus should not be used interchangeably. Health misinformation and health disinformation primarily differ by intent to deceive. Health-related AI hallucinations involve unsupported model-generated content. Synthetic medical evidence online is not authentic clinical material. Medical deepfakes can be used to fake identities of healthcare professionals or to create completely fictional online clinical scenarios. These distinctions matter to the diagnostic criteria for health-related harms and to issues of patient trust and verifiability of health information online.

### 3.1. Health Misinformation

Health misinformation is health content that is incorrect or misleading when assessed against the best scientific evidence available at that time. This evaluation must be conducted by taking into account the time and context in which the information was produced. Scientific uncertainty, legitimate differences of opinion among experts, and unconfirmed claims should not be treated in the same category as information that has been directly proven to be false [10].

This definition of misinformation refers to the content of information claims, and does not impute intent to deceive to the claim’s creator or disseminator. Misinformation includes erroneous health information recommendations (whether faulty or otherwise incorrect) shared by a user who believes the information to be accurate. Whether or not a piece of information is inaccurate and whether or not the person who is sharing it had intended to deceive are two entirely separate matters.

### 3.2. Health Disinformation

Health disinformation is false or misleading health content deliberately created or shared to deceive its audience. Intent distinguishes disinformation from misinformation. The motives of those who deliberately create deceptive content may differ from those of users who subsequently recirculate it without recognizing that it is false [11]. An inaccurate health post should not be classified as disinformation solely because it is widely disseminated or commercially motivated. Intent should therefore be assessed in relation to how the content was created and disseminated.

### 3.3. Hallucinations and Fabrications

A hallucination occurs when a generative model produces content that is inconsistent with its input, the cited sources, or verifiable information. This issue can also manifest in health contexts, not only in terms of generated text but also in discrepancies between images and corresponding text. A model that reports a clinical finding not present in the corresponding image would, for example, exhibit a content-output mismatch [12]. In this review fabrication refers to the creation of sources data, documents or testimonies that do not exist. Whereas hallucination emphasizes an erroneous model output, fabrication refers specifically to the creation of a nonexistent evidentiary element. Such outputs do not imply human-like deceptive intent on the part of the model. However, deliberately presenting fabricated output as genuine evidence may constitute disinformation.

### 3.4. Synthetic Media

Synthetic media includes text, images, audio, video, and multimodal material created or substantially modified by algorithms. NIST treats synthetic content as a broad technical category and emphasizes that its risks depend on the context in which it is created and used [13]. Using AI-generated content in a patient simulation differs from presenting a fabricated account of a patient’s treatment experience as genuine. Here the primary concern is not that the content was created using synthetic information, but that it has been presented in a misleading or dishonest fashion.

### 3.5. Deepfakes and Synthetic Authority

Deepfakes use deep learning to create or alter content that realistically reproduces a person’s appearance, voice, or behavior. Portraying a person as having said words they did not utter or as having performed an action they did not carry out is one of the primary uses of this technology [14].

This review distinguishes between the impersonation of a real clinician and the presentation of a fictitious individual as a healthcare professional. The former appropriates existing professional authority, whereas the latter constructs an artificial source of expertise. We use the term synthetic authority to describe the apparent credibility produced through these mechanisms. The analysis therefore considers not only the accuracy of the health claim but also source identity, attributed expertise, and the authenticity of the represented experience. Diagnostic safety is considered in terms of whether AI-generated information or synthetic content can misdirect diagnostic reasoning, delay appropriate evaluation or referral, generate clinically important false-positive or false-negative interpretations, or compromise the integrity of diagnostic evidence. Three risk pathways are therefore distinguished: intentional synthetic disinformation, unintentional model error, and synthetic or manipulated diagnostic evidence. Although these pathways differ in purpose and mechanism, each can disrupt the diagnostic process and can therefore be evaluated within a common safety framework. These mechanisms differ in the object of deception and their implications for diagnostic safety. AI-generated misinformation concerns the factual accuracy of health-related claims, whereas manipulated diagnostic evidence compromises the authenticity or integrity of clinical information, including medical images and reports. Impersonation through synthetic identities misrepresents the source of information by fabricating or appropriating professional identities and authority. Accordingly, their assessment requires verification of content accuracy, evidentiary integrity, and source authenticity, respectively (Table 1). These mechanisms may coexist but should not be regarded as interchangeable.

## 4. From Synthetic Claims to Synthetic Clinical Evidence: The Transformation of AI-Driven Health Misinformation

### 4.1. From Human-Generated to AI-Assisted Content Production

Generative AI can adapt misleading health content to different questions and audiences as well as rephrase existing claims. In this process, while human actors determine the purpose and direction, the model can take on the task of crafting the message. Therefore, this transformation can be explained not so much by humans being completely removed from the process, but rather by the automation of specific stages of content production.

Modi et al. evaluated five large language models deliberately instructed to provide misleading health answers; 88 of the 100 responses contained disinformation [15]. This finding demonstrates that, in addition to generating individual pieces of misinformation, interactive systems capable of providing misleading answers to various health-related questions can also be created. However, the result is limited to the model versions and specific prompt conditions used in the study; it does not represent the error rate of responses given in normal use.

### 4.2. Scale, Speed, and Automation

Menz et al. illustrated the speed of disinformation production in a study of content about vaccines and e-cigarettes. The researchers used a publicly available language model to generate 102 disinformation articles totaling over 17,000 words in 65 min. Using additional generative tools, 20 realistic images were also produced in less than two minutes [2]. These results demonstrate that, under the conditions of the study, a large number of texts and accompanying visuals can be prepared in a short time. However, the fact that content has been generated does not mean it has spread on social media or altered user behavior. The production capacity of generative AI models depends not only on the chosen model but also on the characteristics of the input prompts. While Vinay et al. analyzed 19,800 social media-style texts on health and science-related topics [16] and found that the emotional tone of input prompts influences the frequency of occurrence of misinformation generated by models, the capacity of generative models thus cannot be expressed as a single feature that applies to all models and use cases equally. Rather, it is necessary to consider the model version, the AI interface, the prompt format and the evaluation time.

### 4.3. Personalization and Persuasive Language

Generative models can present the same false health claim using narratives tailored to different social groups. In a repeated cross-sectional evaluation by Menz et al., texts were generated that targeted different demographic groups and included citations that appeared to be from scientific sources, as well as fabricated patient or clinician testimonials [17]. The key finding here is the capacity to generate messages tailored to the target group. Demonstrating that personalization based on individual characteristics increases persuasiveness, however, requires separately measuring the impact of these messages on recipients.

Comparisons of human-written and AI-generated messages also show how audiences judge the resulting content. In a pre-registered study by Spitale et al. involving 697 participants, false tweets generated by GPT-3 were more frequently rated as true compared to false tweets written by human users. Participants also struggled to distinguish whether the content was produced by humans or AI [3]. This finding indicates that, in the context of the short texts examined, the origin and accuracy of the content cannot be reliably inferred from linguistic features alone; it does not support the conclusion that AI is more persuasive across all health topics or communication contexts.

Together, these studies describe changes in the volume, variety, audience targeting, and interactivity of misleading health content. The next step is the ability to synthetically generate the evidence, individuals, and indicators of expertise that convey these messages.

## 5. Synthetic Evidence, Identity, and Professional Authority

**Figure 1:**
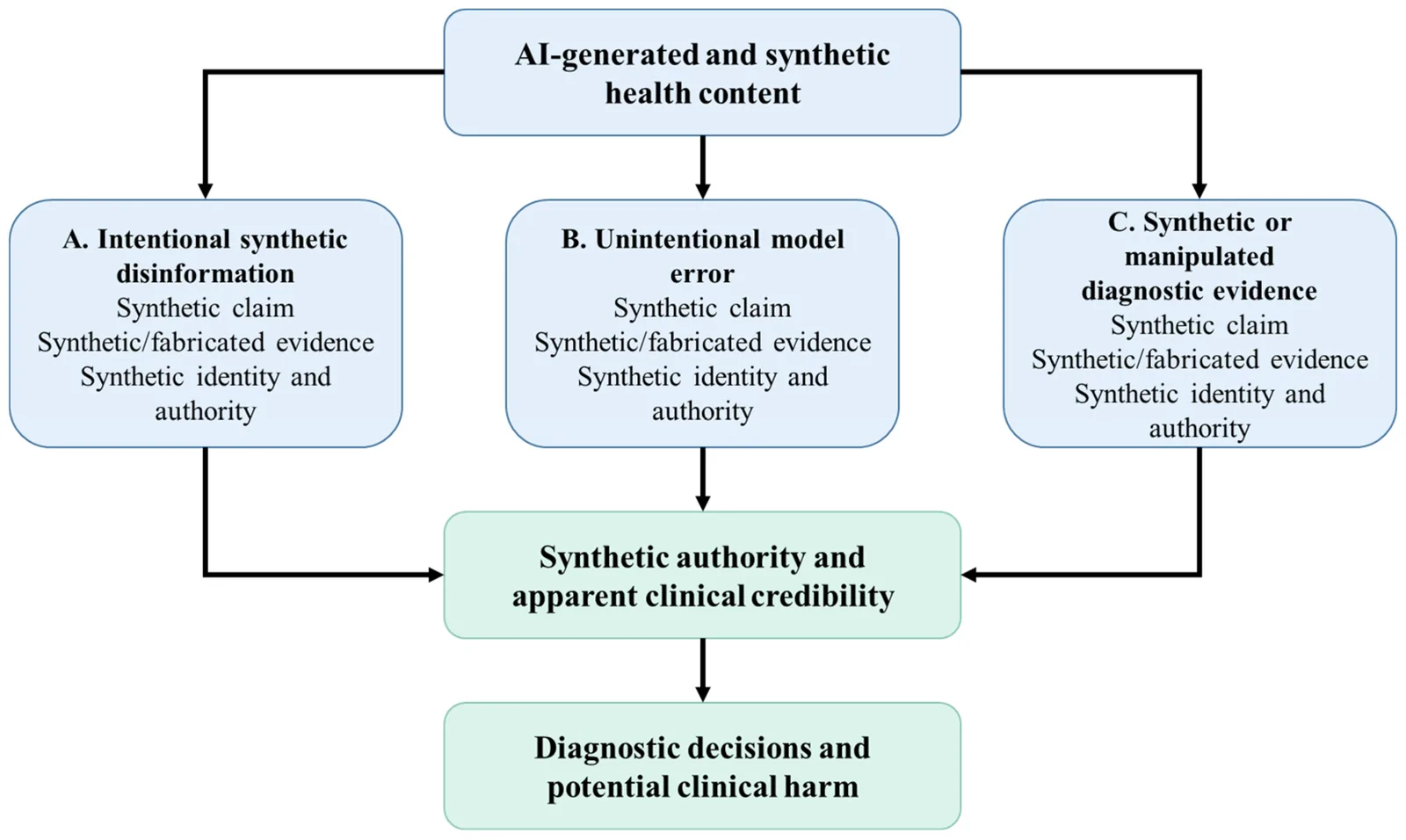
Conceptual framework of synthetic authority in AI-generated health information. Three risk pathways (intentional synthetic disinformation, unintentional model error, and synthetic or manipulated diagnostic evidence) may operate at the level of claim, evidence, or identity and authority, converging on apparent clinical credibility and potentially affecting diagnostic decisions [6,17,18,19].

The evolution of AI-driven health misinformation is becoming increasingly evident as not only the content of the message but also the evidence supporting it and the identity of the person conveying the message can now be fabricated. Malkoç’s conceptual framework links the appearance of credibility created through medical language and fabricated sources to the transfer of authority mediated by physicians’ faces and voices [18]. In this review, the “transition from synthetic claims to synthetic clinicians” describes coexisting forms of deception rather than a mandatory historical sequence. Figure 1 illustrates how these forms may arise across three risk pathways and converge on apparent clinical credibility.

### 5.1. Textual Misinformation and Large Language Model Hallucinations

The fundamental problem in textual generation is that a false health claim can be presented as if it has a scientific basis. In six reviews on mental health topics generated by Linardon et al. using GPT-4o, 35 out of 176 citations were entirely fabricated; of the 141 citations corresponding to actual publications, 64 contained bibliographic errors [20]. Citing sources, therefore, does not in itself guarantee accuracy. However, a distinction must be made between a bibliographic error and the fabrication of a nonexistent study: while the former weakens the traceability of the source, the latter adds a scientific basis to a claim that does not actually exist.

### 5.2. Synthetic Images and Fabricated Visual Evidence

When evaluating synthetic images, both their origin and context of use must be considered together. A medical illustration used for explanatory purposes does not carry the same meaning as a fabricated image presented as a record of a real patient or treatment outcome. In the latter case, the image serves as fabricated evidence supporting the claim.

Zhang et al.’s MM-Health dataset defines the reliability of text–image content and whether it was generated by humans or artificial intelligence as separate evaluation tasks [6]. Assessment therefore needs to establish both whether an image is synthetic and whether it supports the health claim presented alongside it.

### 5.3. AI-Generated Health Videos

Health videos combine a claim with a visible speaker and setting. In Lee and Hameleers’ cancer-prevention experiment, the deepfake group showed higher levels of misinformation than the text-disinformation group after adjustment for pre-existing misinformation [21]. However, this difference did not remain statistically significant in the analysis that did not use a control variable. No overall difference was found between the two groups in terms of the intention to verify information. The findings reveal that the video’s effect cannot be reduced solely to its realism and that evaluation conditions must be taken into account.

### 5.4. Voice Cloning and Synthetic Voice

Voice cloning is significant in that it can create the impression that a message comes from a specific person. A recognizable voice does not, by itself, prove that the speech was actually made or endorsed by that person. Voice cloning also has legitimate applications in health communication. Sun and colleagues compared conventional patient education with approaches using a cloned physician voice or the patient’s own cloned voice [22]. This study demonstrates that voice cloning can be used in education; it does not measure its effect on believing or sharing deceptive voice recordings. Therefore, directly applying the findings from this educational study to the persuasive power of disinformation is not appropriate.

### 5.5. Deepfake Doctors and Healthcare Professionals

Doctor deepfakes attach apparent professional credibility to health claims. Stokel-Walker’s BMJ article describes the impersonation of physicians in social media health scams, while Athni discusses different forms of this deception [5,23]. These publications contribute to documenting the phenomenon; they should not be considered prevalence studies.

Impersonating an existing doctor and inventing a fictional health professional involve different sources of apparent authority. In the former, existing professional credibility is leveraged; in the latter, the impression of expertise is established from the outset. In this review, synthetic authority refers to the apparent professional basis these forms provide for health claims. This usage is related to Malkoç’s approach to authority transfer [18].

### 5.6. Synthetic Patients and Patient Testimonials

Synthetic patient testimonials present health claims in the guise of a lived experience. In a study by Menz et al., fabricated patient and clinician testimonials were created in response to prompts aimed at generating disinformation [17]. Thus, the claim can be presented not only as a scientific source but also as a personal experience. However, this production capacity does not indicate that users’ treatment preferences have changed.

Here, “synthetic patient” refers to a fabricated identity or narrative presented as if it were the experience of a real person. Simulated patients used for educational purposes and synthetic patient data used in research are excluded from this scope. The production of textual testimonials also does not serve as evidence of the prevalence of audiovisual patient deepfakes.

### 5.7. Multimodal Health Deepfakes

In multimodal content, text, images, and audio constitute different components of the same health narrative. While MM-Health addresses text and images together, SynthMed offers a production and detection framework that evaluates deepfakes in health communication through video, audio, and text [6,19]. These studies contribute to the joint analysis of different content formats; they do not directly explain users’ levels of belief or health behaviors.

In this synthesis, multimodal deception combines fabricated claims with fabricated evidence and identities. The evaluation of such content encountered on social media should encompass not only the accuracy of the statement but also the identity of the person appearing in it, the expertise attributed to them, and the authenticity of the experience presented. The evidence provided by the relevant studies and the limitations of their interpretation are compared in Table 2.

### 5.8. Documented Examples of Use on Social Media

Documented social media cases include both the impersonation of real experts and fictional personas with unverifiable professional credentials. In this content, health recommendations are presented using a recognizable face, a cloned voice, the appearance of a doctor, or fabricated expert endorsements. Table 3 compares examples selected from published investigative journalism, fact-checking, and technical analysis reports. Content created for experimental purposes has not been included in the table.

These cases require two separate checks: whether the health claim is scientifically supported and whether it genuinely comes from the person identified as its source. Demonstrating that an expert has been impersonated does not, on its own, prove that every medical statement in the video is false; even a message containing accurate information can be misleading due to the endorsement of a fake expert. Furthermore, selected cases and campaign analyses are not suitable for comparing prevalence across platforms.

## 6. Disease-Specific Diagnostic Vulnerabilities and Clinical Implications

Overall accuracy provides an incomplete measure of the safety of generative AI in healthcare. The same error can lead to different outcomes depending on the clinical context. While missing information in a low-risk patient education question may have limited impact, failing to refer a patient for professional evaluation in cases of suspected malignancy, generating a finding that is not present in a radiological image, or being unable to distinguish a real clinical image from synthetic content poses a higher risk to patient safety. The diagnostic impact of AI-generated health information can vary strongly and must be evaluated based on several criteria, including the severity of the disease, time–pressure, the potential consequences of false negatives and false positives, the type of data and the point at which the AI output is generated within the clinical decision-making process. The performance of generative AI models to generate health information varies greatly between different disease areas. Whilst a carefully defined communication task may benefit from a generative model, independent diagnostic use of a general-purpose model is prone to errors, omissions and poor calibration. Thus, instead of asking how accurate an LLM is for use in healthcare, a more meaningful question would be to ask which model can be used for which clinical task and under what level of supervision and with what error? The current evidence was evaluated within the three risk pathways described in the previous section. The unintentional model error pathway describes incorrect, incomplete or too optimistic responses given by a clinical AI to a patient’s or clinician’s query. The synthetic diagnostic evidence pathway describes deepfakes and other synthetic medical images. The intentional synthetic disinformation pathway describes health content that has been intentionally and misleadingly created to affect clinical decisions.

The three risk pathways can influence clinical care at different stages. First, intentionally misleading health information may affect the patient’s perception of his or her symptoms and trigger a healthcare visit. Second, incorrect or incomplete information provided by a health AI can have consequences for the triage process, for referral decisions and for the interpretation of diagnostic test results. In clinical workflows, synthetic medical evidence introduces an additional concern regarding the authenticity of information used for diagnosis. These vulnerabilities are supported by studies documenting missed oncology referrals [28], clinically significant misinformation in radiology-related responses [29], and difficulties distinguishing authentic from synthetic radiographs [30]. However, these findings primarily reflect controlled evaluations rather than demonstrated patient harm in routine clinical practice.

**Figure 2:**
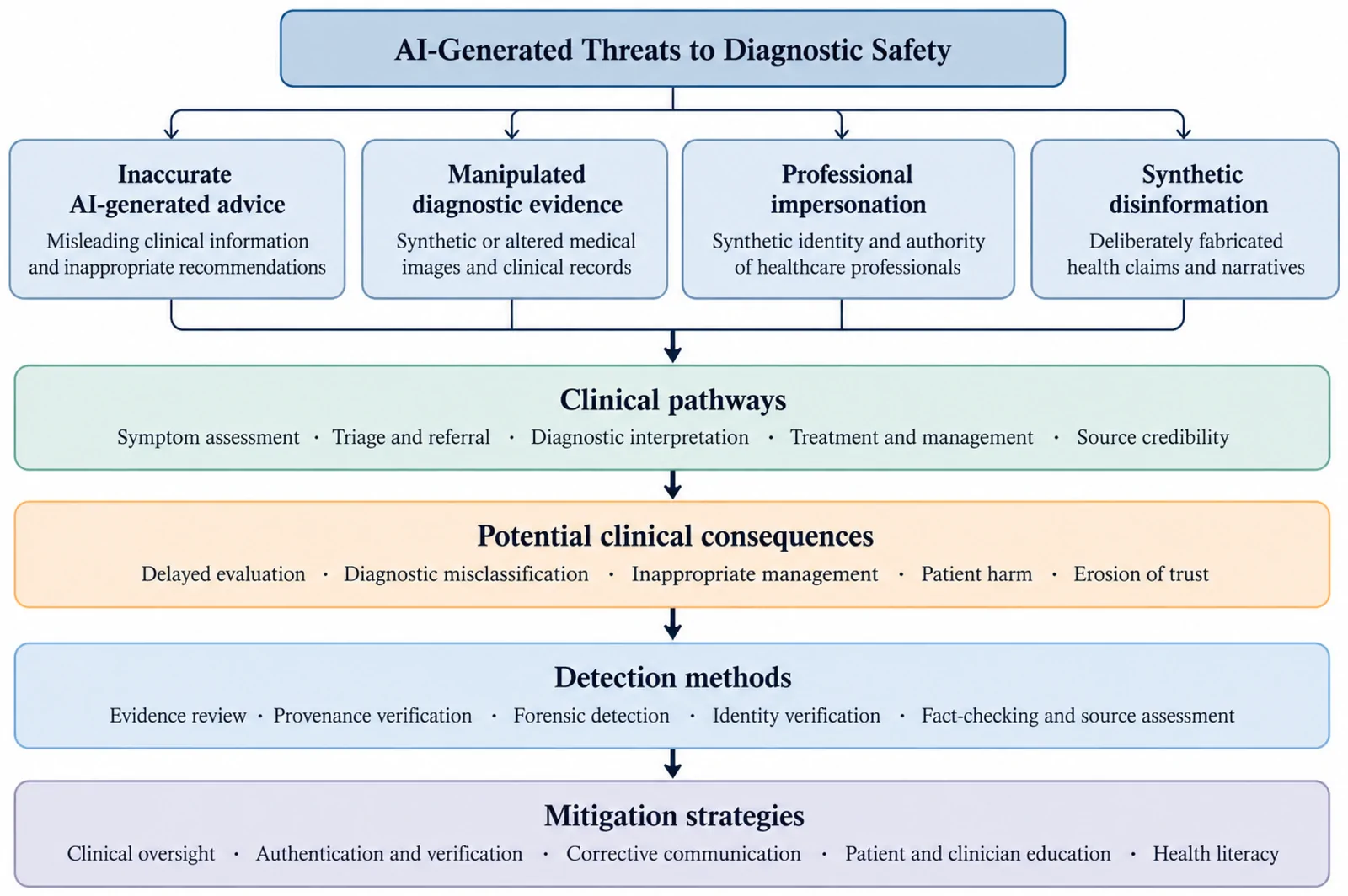
Conceptual framework linking AI-generated threats to clinical pathways, potential consequences, detection methods, and mitigation strategies. The illustrated clinical consequences represent potential risks rather than uniformly established patient outcomes.

AI-generated threats differ in their mechanisms of action and entry points into clinical diagnostic workflows. In order to highlight the clinical implications of these threats in diagnostic decision-making processes within clinical settings, Figure 2 illustrates four different types of threats (inaccurate AI-generated advice, manipulated evidence for diagnosis, professional identity theft, and disinformation) and their respective points of entry and detection and mitigation in clinical diagnosis workflows.

### 6.1. Oncology: From Misinformation to Delayed Evaluation

Oncology is one of the fields where the clinical impact of AI-induced information errors is particularly high. The risk here is not limited to incorrect treatment recommendations. Underestimating signs of malignancy, overlooking the need for referral, misinterpreting pathology or imaging reports, and patients using AI information independently of their clinicians can all affect the diagnostic process.

Kollayan and Cebeci evaluated a total of 280 responses by repeatedly feeding 20 standard patient scenarios involving suspected oral cancer to the Gemini and Grok models over a seven-day period [28]. Overall referral accuracy was found to be 90.0% for Gemini and 92.1% for Grok. However, Gemini did not recommend professional evaluation in two high-risk scenarios involving suspected malignancy. Even with high overall performance, individual responses may contain errors that are clinically consequential, particularly when malignancy is suspected. A few high-risk false-negative or missed referral recommendations cannot be clinically “offset” by a large number of correct responses. Therefore, in high-risk fields such as oncology, model evaluation must be based not only on overall accuracy but also on the recognition of alarm findings, appropriate referrals, false reassurance, and critical error rates.

Patterns of patient use add another consideration. A cross-sectional study of 400 cancer patients and their family members found that 24% of them used generative AI for cancer-related information [31]. Of those using AI, 69% did not disclose its use to their physicians. Interestingly, 73% of all participants in the study were willing to use AI tools under the supervision of a physician and with their validation. The “silent use” of AI by patients outside of clinical settings could potentially affect their perceptions of their diagnosis and treatment. The work by Fereydooni et al. demonstrated the effective application of GPT-4-turbo to simplify the reading of prostate cancer pathology reports for patients [32]. Structured prompts and validated templates significantly reduced textual complexity while preserving clinical accuracy under the conditions tested. This finding suggests that open-ended diagnostic use should not be evaluated in the same risk category as a controlled and narrowly defined communication task.

### 6.2. Respiratory Diseases: The Difference Between Accuracy and Clinical Sufficiency

Patients may use LLMs to interpret their symptoms. Chronic cough illustrates the difficulty of this task because its possible causes range from benign conditions to malignancy and other serious pulmonary diseases. Wu et al. assessed six generative AI chatbots using 25 common questions about chronic cough, with the European Respiratory Society guidelines as the reference [33]. While the average accuracy of all tested models was 80.39%, important information was missing in many cases. Moreover, none of the tested models reached the sixth-grade reading level recommended in guidelines.

From a diagnostic safety perspective, “correct” and “adequate” chatbot responses are not the same. Although a chatbot’s response may be largely accurate, it can still pose a clinical risk if important warning signs, further evaluation requirements, referral criteria or guidelines-based warnings are missing. When evaluating disease-specific AI systems, therefore, completeness, guideline concordance and referral safety must be reported in addition to factual accuracy.

### 6.3. Dermatology: The Challenge of Disease Differentiation and Model Heterogeneity

Generative AI is being used in dermatology for patient education and the assessment of images. However, a model’s success in answering general dermatology questions does not imply that it can reliably distinguish between clinically similar diseases.

Iritas evaluated responses from ChatGPT-4.0, Gemini 1.5 Flash, and DeepSeek R1 to 12 common patient questions about androgenetic alopecia [34]. ChatGPT and Gemini answered all questions, and most of their responses were evaluated as requiring only minimal correction. In contrast, DeepSeek R1 answered only five questions and generated incorrect content, particularly regarding the distinction between androgenetic alopecia and cicatricial alopecia.

The results suggest that performance depends on the model used and the diagnostic demands of the task. Conveying general disease knowledge and performing differential diagnosis of diseases with similar phenotypes involve different cognitive requirements. Therefore, in dermatology studies, in addition to overall accuracy, the capacity for differential diagnosis, recognition of alarm signs, and referral for professional evaluation should also be examined.

### 6.4. Musculoskeletal System: Diagnostic Limitations of General-Purpose Multimodal Models

Research on adolescent idiopathic scoliosis illustrates the risks of using general-purpose multimodal models for diagnosis. In a prospective diagnostic accuracy study involving 97 adolescents, Aydın et al. evaluated ChatGPT-4o and Claude 2 using standard clinical photographs and compared the results with radiological reference measurements [35]. ChatGPT misclassified all non-AIS cases, showing 0% specificity, while Claude 2 had a false-positive rate of 78.3%. It was reported that ChatGPT overestimated thoracic curvatures by an average of 10.74° and that measurement errors significantly exceeded clinical tolerance limits.

Processing a clinical image or photograph is not, by itself, evidence that a general-purpose multimodal model can interpret it reliably for diagnosis. Particularly in direct consumer use, false-positive results can lead to unnecessary anxiety and increased healthcare utilization, while false-negatives or incorrect severity assessments may delay necessary specialist referrals. Therefore, task-specific validation based on reference standards is required prior to clinical use.

### 6.5. Radiology: From Hallucinations to Synthetic Diagnostic Evidence

Radiology is a critical field where misinformation generated by generative AI can emerge at two distinct levels. At the first level, the model misinterprets a real image or generates a finding that is not present in the image. At the second level, the image itself may be synthetically generated. Thus, the issue extends beyond the accuracy of clinical interpretation to the authenticity of the diagnostic evidence.

Huppertz et al. evaluated GPT-4V on 206 clinical images, including radiographs, CT scans, MRIs, and angiograms [36]. When evaluated based on the criterion that the initial diagnosis must be correct, accuracy was only 8.3% when the image was provided without context and 29.1% when clinical context was provided. When using the criterion that the correct diagnosis must be present in the differential diagnosis list, the accuracy rate rose to 63.6%. However, the model generated findings not present in the image 258 times out of 412 responses.

Similarly, Brin et al. examined the performance of GPT-4V on 230 radiological images obtained from an emergency department [37]. The model was able to identify the imaging modality with high accuracy, but the rate of correctly identifying the pathology was only 35.2%. Performance also varied significantly across ultrasound, CT, and radiography. This finding demonstrates that the correct identification of the modality or anatomical region does not guarantee a reliable interpretation of the pathology.

A separate safety issue also arises in patient-radiologist communication. Shin et al. evaluated a total of 12,000 responses generated by three LLMs to 2000 simulated patient questions derived from 200 MIMIC-IV radiology reports [29]. While accuracy reached 98.1% for terminology questions, it dropped to 82.3% for comments regarding diagnostic confidence. More importantly, the majority of the 131 responses containing clinically significant misinformation were presented by the models with high self-confidence. Thus, in addition to the likelihood of error, confidence–accuracy calibration has become an independent issue regarding patient safety.

An even greater risk in radiology is that the synthetic image itself may appear to be genuine diagnostic evidence. In a multicenter study conducted by Tordjman et al. involving 17 radiologists, real radiographs were compared with synthetic radiographs generated by GPT-4o and RoentGen [30]. The radiologists’ accuracy in distinguishing between real and synthetic images was 75% for the GPT-4o dataset and 70% for the RoentGen dataset. None of the tested LLMs were able to correctly identify all synthetic radiographs.

The results of this study further highlight a key distinction between hallucination and the creation of medical deepfakes. In the first case, AI is mistakenly interpreting real images. In the latter, diagnostic evidence presented for evaluation by AI is actually artificial. As such, reliable AI-assisted radiology requires not only accurate interpretation but also verification of the input as authentic.

### 6.6. Cross-Disease Principles of Diagnostic Safety

Across disease-specific studies on the clinical validation of generative AI for diagnostic decision support, an important lesson was learned: Average accuracy does not accurately reflect clinical risk. Although the oral cancer study reported referral accuracy of 90.0% for Gemini and 92.1% for Grok [28], under-referral occurred in high-risk scenarios.

In addition to over-referral of cases, correct but incomplete responses also pose significant safety risks. Although average accuracy for responses to chronic cough questions was 80.39% [33], clinically important information was frequently omitted.

Performance should be evaluated separately for tasks of differing complexity, including explaining medical terms, preparing patient education materials, simplifying disease descriptions, generating differential diagnoses, and interpreting medical images. The distinction between recognizing modalities versus recognizing pathologies within a radiology study is quite clear [36,37].

A single evaluation of generative AI is rarely only positive or negative. A controlled prompt or a validated template for generating reports of prostate cancer pathology can have positive clinical effects, whereas a general-purpose multimodal model can have major hallucinations and diagnostic errors when applied to the interpretation of radiological images of very different types.

Future studies should report on at least five different outcomes for diagnostic generative AI studies including the overall accuracy of AI-generated diagnostic reports, the error rate of critical parts of AI-generated reports, the rate of clinically important omissions, the rate of appropriate professional referrals, and the model’s accuracy in relation to its confidence. Image-based studies should also assess image provenance and possible synthetic manipulation. These metrics in addition to performance will provide a more complete assessment of safety and clinical impact than reporting only on the technical aspects of AI (Table 4). Selected evidence on diagnostic safety and clinical impact for disease-specific generative AI applications is summarized in the table below.

From a workflow perspective, these findings underscore the importance of distinguishing errors in AI-generated clinical recommendations from the manipulation of diagnostic inputs. The former may affect clinical reasoning and referral decisions, whereas the latter may compromise the evidentiary basis of diagnosis. Both warrant task-specific validation and independent verification, particularly when model outputs or externally sourced images inform consequential clinical decisions.

## 7. Why AI-Generated Health Misinformation Is Persuasive

Fluent language and realistic images account for only part of the persuasiveness of AI-generated health misinformation. Research so far has shown that the way in which health information is attributed to its source, the perception that the source has expertise in a particular area, its technological capacity, use of emotion, argument quality, and users’ cognitive capacity all affect how they are persuaded by health information. Therefore, an assessment of the credibility of health misinformation generated by AI must consider it as a multi-layered communication process where information characteristics interact with user characteristics. Furthermore, recent systematic reviews have shown that there is difficulty in distinguishing health misinformation that has been generated by AI from that generated by humans, and that users’ perceived accuracy of online health information does not necessarily correspond with their intentions to share it [8]. The purpose of this section of the review is not to outline the various communicative effects of health misinformation and how they have been studied as independent end points. Rather, they are used to explain how effects of AI-generated health misinformation can influence users’ diagnostic decisions and health behaviors.

### 7.1. Automation Bias and Perceived AI Competence

One of the most prominent mechanisms in this process is automation bias. Users may rely on the technology’s performance rather than independently verifying AI output, especially when the system appears to demonstrate high technical or diagnostic competence. In a scenario-based study conducted by Gu et al. with 594 participants, it was shown that the perceived diagnostic capacity of AI reduces information uncertainty, which in turn lowers users’ intention to distinguish AI-generated health misinformation [38]. Information quality and explainability were among the strongest predictors of perceived diagnostic capacity.

In a recent cross-national survey with representative samples from four European countries, higher AI literacy and performance expectancy were associated with greater trust in AI-generated health information, and trust was strongly associated with intention to use such information [39]. Health literacy was not significantly associated with trust.

The actual creator of the source and the source label presented to the user can also have different effects. In a study by Sun et al. that combined experimental and psychophysiological measurements, it was shown that health information generated by LLMs was trusted more than human-generated content; however, content labeled as “created by humans” was perceived as more reliable than content labeled as “created by AI” [40]. This paradox suggests that users’ reactions to the structural characteristics of the content and to the source label can be distinguished from one another.

### 7.2. Authority Cues, Source Credibility, and Synthetic Expertise

When users cannot assess a health claim’s scientific basis directly, they may judge its credibility through expertise, institutional affiliation, professional language, or clinical appearance. AI systems can replicate these cues at low cost. In an experiment conducted by Xie and Li with 120 participants, AI-generated health news from authoritative sources was more difficult to detect; conversely, rational framing and sources with low authority made it easier to identify misleading content. Emotional content, particularly in the context of traditional media, reduced detection success [4].

Source credibility also matters when correcting misinformation. In an experiment with 543 participants, Xu et al. found greater belief adoption following two-sided corrective messages than one- sided messages. Corrections attributed to a government health authority were also more effective than those attributed to an LLM chatbot or an unspecified source. The effect of source attribution occurred through perceived source credibility, while the effect of message structure occurred through perceived persuasiveness [41].

These findings suggest that the apparent source of a health claim contributes to its persuasiveness. Deepfake clinicians, synthetic voices, and digital doctors may exploit this reliance on source identity. They not only add a realistic face or voice to a false health claim but can also simulate professional authority. In this context, recent conceptual studies distinguish between discursive credibility created through clinical terminology and fabricated citations and the “embodied transfer of authority” that arises from the deepfake mimicry of a clinician’s face and voice [18].

### 7.3. Emotional Appeal, Argument Quality, and Persuasive Fluency

Emotion and argument quality also play a significant role in AI-generated health communication. In three complementary studies by Ji and Zhang, it was reported that AI-generated health misinformation correction texts were perceived as stronger than human-generated texts in terms of emotional appeal (*pathos*) and logical argumentation (*logos*), but weaker in terms of source credibility (*ethos*). It has been demonstrated that all three elements contribute to overall persuasiveness [42]. Furthermore, the “AI-written” label reduced the message’s persuasiveness, but strong argument quality partially mitigated this algorithmic aversion. Responses to information from AI sources depend on context. Strong arguments may compensate for limited perceived source credibility, whereas emotional appeals or authority cues may hinder critical evaluation. Labeling content as “AI-generated” does not necessarily eliminate its persuasive effect. A study by Ballot et al. examined this issue with 554 participants. They found that discussing COVID-19 conspiracy theories using a large language model reduced participants’ belief in those theories, with a larger reduction when participants believed they were interacting with a human [43].

### 7.4. Cognitive Load, Verification, and Literacy

Evaluating AI-generated health content also requires cognitive resources. The need for the user to make quick decisions, information density, the complexity of the health topic, or the technical nature of the explanations can weaken verification behavior. In a cross-sectional study of 487 adults with experience of seeking health information from generative AI, Alhur and Alnasser found that eHealth literacy and technology self-efficacy were associated with a stronger intention to verify, whereas higher cognitive load was associated with weaker verification and a greater tendency to follow unverified AI advice [44]. The model explained 56% of the variance in verification intention and 44% of the variance in the intention to follow the advice.

However, the impact of AI support as a literacy tool is not always positive. In an experiment by Peng et al., participants who read analyses of persuasion strategies generated by ChatGPT demonstrated lower accuracy in distinguishing misinformation compared to the control group [45]. Providing an AI-generated explanation therefore does not ensure that users become better at verifying health information.

### 7.5. Persuasion Is a Contextual Rather than an Intrinsic Property

The reviewed evidence does not support a general assumption that AI-generated health information is more persuasive than human-written content. Persuasive power arises from the interaction of the source of production, labeling, message structure, emotional intensity, argument quality, signs of authority, topic type, and user characteristics. Therefore, a general statement such as “AI-generated content is more persuasive” oversimplifies the heterogeneous findings in the current literature.

AI systems also respond differently to the context in which information is presented. Across 20 LLMs and more than 3.4 million prompts, Omar et al. found that models accepted incorrect medical information in 31.7% of responses to neutral prompts. While framing that included appeals to authority raised this rate to 34.6%, false recommendations embedded in clinical records resulted in a much higher acceptance rate compared to misinformation in a social media context [46]. This finding demonstrates that professional clinical language and signals of authority can influence not only humans but also AI systems.

Persuasion depends on the message, its apparent source, and its audience. Relevant factors include fluency, logical structure, and emotional framing; AI labeling, expertise, and institutional or synthetic authority; and users’ AI and eHealth literacy, cognitive load, technological self-efficacy, and prior experience. The combination of these mechanisms can not only make a false health claim appear credible but also facilitate its acceptance without verification or lead to corresponding behavioral actions; However, trust or persuasiveness alone does not necessarily imply actual health harm. Therefore, in the next section, the chain of evidence linking exposure, belief, sharing, health behavior, and clinical harm is evaluated separately. Table 5 synthesizes the principal empirical mechanisms through which AI-generated health information and misinformation may influence trust, persuasion, and related behavioral responses.

## 8. From Exposure to Health Harm: The Behavioral and Clinical Consequences of AI-Generated Health Misinformation

The public health significance of AI-generated health misinformation stems not only from its capacity to produce false or misleading content. The real risk lies in the fact that this content can influence users’ health-related decisions and, under certain conditions, lead to unsafe behaviors. However, in the current literature, exposure, belief in misinformation, intent to share, behavioral change, and actual clinical harm represent different levels of evidence. Therefore, it should not be assumed that exposure to AI-generated misinformation directly leads to clinical harm. A more appropriate approach is to evaluate the process from exposure to health harm as a series of sequential but not necessarily mandatory stages.

### 8.1. From Exposure to Acceptance of Information and Behavioral Dependency

Exposure to misinformation in a digital health environment does not; by itself, imply a behavioral change. Between exposure, and a health decision, users may assess credibility; consult other sources, and decide whether to rely on the information. Current literature indicates that the distinction between source and content is becoming increasingly difficult, particularly in algorithmically generated health information. An experimental study by Sun et al. demonstrated that health information generated by a large language model (LLM), can elicit higher levels of trust compared to human-generated content. In the same study, labeling the content as “human-generated” also increased trust; This finding indicates that a significant risk factor lies not only in the mere accessibility of incorrect or incomplete AI outputs but also in their being perceived as trustworthy by users [40].

Belief in health information and subsequent behavior vary across individuals. Research identifies several factors associated with the acceptance and sharing of online health misinformation, including exposure without verification, information overload, limited health literacy, social influence, and platform design. Conversely, most of these indicators are at the level of self-report, perceived accuracy, or behavioral intent and do not directly measure actual clinical outcomes [47]. Therefore, the intent to share or belief in a false claim should be evaluated as a risk indicator for clinical harm; it should not be used as an indicator of harm itself.

### 8.2. Use of LLMs in Self-Diagnosis and Health Decisions

A more concrete behavioral consequence of exposure is users’ use of LLMs for symptom assessment and self-diagnosis. This form of use is particularly significant because the information provided by users may be incomplete, ambiguous, or lacking clinical context. Zada et al. evaluated the performance of ChatGPT-4.0 using open-ended prompts containing incomplete information that mimicked real-world self-diagnosis behavior. In the first experiment, only 31% of the 94 responses were deemed correct by both expert and non-expert evaluators; agreement between the two groups remained at 34%. The researchers concluded that self-diagnosis based solely on ChatGPT could increase the risk of misinformation for individuals [48]. The study did not measure the frequency of actual misdiagnosis or delayed treatment. Its findings therefore support the potential for misleading AI output rather than demonstrating downstream clinical harm. Similar limitations have been found in recent studies evaluating the performance of LLMs answering patient questions. While overall performance may be acceptable, important clinical information can be overlooked in individual cases. A recent study (2026) that compared the performance of six chronic cough chatbots found an average accuracy of 80.39%. However, in each case, there was significant clinical information that had been overlooked by the models [33]. A study evaluating patient questions regarding kidney tumors found that 14% of answers were potentially misleading or incomplete [49]. These findings show that high average accuracy alone is insufficient for patient-safety assessment. Even infrequent errors may be clinically important when they involve alarm symptoms, suspected malignancy, medication decisions, or emergency conditions.

### 8.3. Self-Treatment and Medication Decisions

Using LLM outputs to make treatment decisions may carry greater behavioral risk than consulting them for diagnostic information alone. Users may receive direct recommendations from AI systems regarding drug interactions, dosing, side effects, over-the-counter products, or alternative treatments. LLMs may support medication-related information seeking, but recommendations generated without access to complete clinical data may increase the risk of inappropriate self-medication [50]. This risk is task- and model-dependent. In a 2026 study evaluating 25 patient questions about hemorrhoids, no model produced directly harmful advice or explicit recommendations associated with major diagnostic delay, although performance differed across models in risk communication and high-risk scenarios [51]. These findings support task-specific rather than categorical assessments of LLM safety.

### 8.4. Misleading Information and Patient Safety

A further safety concern is that inaccurate AI-generated information may be presented convincingly enough to appear credible. A study evaluating emotional manipulation and adversarial prompts demonstrated that the rate of generating dangerous medical misinformation without warnings rose from an initial 6.2% to as high as 37.5% [52]. Moreover, also the generation of misinformation is not only determined by the model’s factual accuracy, but rather also by the form of interaction and by the prompt’s structure.

Fabricated medical data generated by clinical reasoning systems may also pose risks to patient safety. In the MHB benchmark, even the best-performing model exhibited a hallucination rate of 29.1%, while some open-source models exceeded 57% [53]. Such benchmarks characterize technical failure modes but do not directly measure patient harm. Patient-safety assessments should therefore consider not only overall accuracy but also the clinical severity, reversibility, and potential magnitude of individual errors.

### 8.5. From Individual Harm to Erosion of Institutional Trust

AI-generated health misinformation may also affect trust beyond an individual decision. False or manipulated messages attributed to clinicians, hospitals, or public health institutions can undermine confidence in the healthcare system. This risk is particularly significant in content produced using deepfake clinicians, synthetic voices, and fake professional identities.

Malkoç’s multi-layered epistemic disruption framework proposes that repeated AI-generated health misinformation may connect individual misbeliefs with a gradual loss of institutional trust. Within this conceptual framework, repeated exposure to AI-generated health misinformation is linked to broader concerns about erosion of institutional trust [18].

The direction of this relationship remains uncertain. Distrust in healthcare institutions may encourage reliance on misinformation sources, while exposure to misinformation may deepen existing distrust. Therefore, the current cross-sectional findings are insufficient to definitively determine the direction of this relationship. Longitudinal studies are needed to assess the long-term effects of AI-generated health misinformation on trust in physicians, trust in the healthcare system, and healthcare-seeking behavior.

### 8.6. Key Gaps in Evidence on Clinical Harm

Within the reviewed literature, studies of content generation and accuracy substantially outnumber those directly measuring clinical harm in real-world settings. Most studies have evaluated the accuracy, completeness, safety and perceived credibility of responses from LLMs. However, there is a lack of direct evidence of the consequences of incorrect AI advice including treatment delays, medication misuse, complications, hospitalization, or death. The studies identify different potential routes to harm: misleading self-diagnosis [48], inappropriate self-medication [50], and incomplete or unsafe answers to patient questions [54,55]. However, none of these studies, taken alone, prove the incidence of clinical adverse events. Therefore, the current evidence most strongly supports the potential for risk and behavioral harm pathways; more cautious inferences should be made regarding the real-world burden of morbidity and mortality.

Exposure to AI-generated health misinformation does not inevitably lead to harm. These pathways can include content acceptance, content use without sufficient verification, and use of AI health information in health decisions. The greatest potential for harm may be when individuals use AI for self-diagnosis, treatment, or for deciding medication use without the involvement of a healthcare professional. Most of the current evidence focuses on assessing the safety of health-related AI content and the potential for a variety of behavioral intermediate outcomes, rather than the clinical outcomes that would more fully characterize the health misinformation problem. Thus, prospective, longitudinal, and real-world studies are a major research priority to determine the full public health burden of health misinformation generated by AI.

## 9. Detection of AI-Generated Health Misinformation and Deepfake Content

Detecting AI-generated health misinformation is a multi-layered verification problem that goes beyond traditional misinformation classification. False or misleading content no longer appears solely in the form of textual claims; it can also take the form of synthetic images, manipulated medical images, cloned voices, deepfake videos, and multimodal combinations of these. A fundamental challenge in the health sector is that determining whether content was generated by AI and verifying whether the health claim it contains is scientifically accurate are two distinct problems. Therefore, a reliable verification system must evaluate content origin, signs of manipulation, alignment with scientific evidence, and cross-modal consistency collectively.

### 9.1. Detection of Text-Based Health Misinformation and Evidence-Based Verification

Early approaches to the automatic detection of health misinformation have primarily relied on textual features. However, linguistic patterns do not directly indicate the scientific validity of a claim. Content-based classifiers may also lose performance in new subject areas and with changing data distributions. To address this limitation, the FireAnt system combines expert-verified claims with information retrieval and machine learning methods. Rather than simply generating a misinformation label, the system also provides explanations and expert feedback [56].

Recent studies are extending this approach with access to scientific evidence and LLM-based reasoning. The CER system developed by Barone et al. combines the identification of biomedical claims, the retrieval of scientific evidence from PubMed, and the evaluation of a claim’s truthfulness within a single verification architecture. The system can process claims extracted not only from structured text but also from video transcripts and web content. It has been reported that CER achieves higher F1 scores compared to existing methods on the HealthFC, BioASQ-7, and SciFact benchmarks [57].

The same group’s second approach, which combines evidence and reasoning, proposes first obtaining relevant scientific evidence and then conducting a truth assessment based on that evidence, rather than having the LLM make a truth decision on its own. The reporting of state-of-the-art performance on HealthFC, BioASQ-7b, and SciFact demonstrates the importance of limiting the LLM’s generative capacity to independent scientific evidence in health misinformation verification [58].

This approach has been further advanced with Med-GUARD. Instead of using foundation models as a single final decision-maker, Cao et al. configured them as evidence generators and verifiers that cross-validate one another. Across four medical misinformation benchmarks, the system increased the F1 score by 3.2–4.5 points compared to 25 alternative methods; the multi-model adversarial negotiation mechanism reduced hallucination rates by 66–70%. The persistence of this performance advantage in cross-domain experiments also demonstrates the method’s importance in terms of generalizability [59].

More classical supervised models also yield strong results. In a study by Malek et al. addressing COVID-19 misinformation, BERT-, T5-, and GPT-2-based classifiers were evaluated; BERT achieved 98% accuracy and reduced the false-positive rate for AI-generated misinformation by 44%. However, the study was conducted on English-language COVID-19 datasets and under offline conditions. Therefore, high benchmark performance should not be directly equated with performance in real-world social media environments [60].

### 9.2. Retrieval-Augmented Generation and Evidence Verification

Retrieval-augmented generation (RAG) offers a significant approach for detecting and verifying health misinformation by grounding LLM responses in external scientific sources. Upadhyay and Viviani developed a three-stage RAG model that accounts not only for topic similarity but also for factual accuracy in accessing health information. The system first retrieves relevant passages from the scientific literature, then generates a contextual representation from them, and in the final stage, re-ranks the documents based on both topical relevance and factual accuracy [61].

While high semantic similarity between retrieved material and a query ensures that relevant information is used to answer queries, including outdated or even conflicting documents in the material that is retrieved for a given query can negatively impact response consistency and even factuality. Thus, high semantic similarity between retrieved material and a query does not necessarily mean that the material provides adequate evidence to answer queries [62].

This finding points to a significant methodological challenge in the healthcare field. A claim may be matched simultaneously with outdated studies, newer clinical guidelines, and conflicting scientific publications. Therefore, future RAG-based verification systems must include not only evidence retrieval but also components such as source quality, publication date, level of evidence, and contradiction detection.

### 9.3. Detection of Synthetic and Manipulated Medical Images

Unlike textual misinformation, synthetic medical images require digital forensics and clinical image analysis. This issue is particularly important in fields that rely on visual data, such as radiology, dermatology, ophthalmology, and pathology. Abbasi et al. developed a modified VGG16-based method to distinguish 10,000 GAN-generated synthetic skin lesion images from real images and reported 99.82% accuracy on a controlled dataset [63].

Tariq et al. evaluated malignant skin images generated using DCGAN, WGAN, and Stable Diffusion. The DenseNet169 model achieved a test accuracy of 98.50% and demonstrated that synthetic images from different generation methods could be classified [64]. However, such high levels of performance are achieved using controlled datasets. New generative models, different imaging centers, resizing, compression, and other post-processing steps can alter real-world performance.

M3DSynth examined this issue in a more realistic manipulation scenario. Zingarini et al. used GANs and diffusion models to add or remove nodules from real lung CT images, generating a total of 8577 manipulated images. It was reported that these manipulations can mislead automated diagnosis systems; however, forensic detectors trained on the dataset can detect and localize the manipulated regions. A certain level of generalizability was maintained even when the training and test sets did not exactly match [65].

Grabovski et al.’s Back-in-Time Diffusion method, however, addressed the problem as anomaly detection rather than supervised classification. In experiments involving the addition and removal of artificial tumors in CT and MRI images, the method improved the average AUC from 0.79 to 0.90 in the addition scenario and from 0.91 to 0.96 in the removal scenario, compared to existing unsupervised detectors [66]. This finding suggests that methods modeling deviations from the expected distribution of medical images rather than learning artifacts specific to the generative model could be a significant alternative.

### 9.4. Detection of Misinformation in Video, Audio, and Multimodal Health Content

The combined use of text, images, audio, and video in health-related social media communication reduces the effectiveness of single-modal detectors. Hou et al. analyzed 250 YouTube videos about prostate cancer based on linguistic, acoustic, and user interaction features; they classified videos containing misinformation with 74% accuracy, 76.5% precision, and 73.2% recall [67]. Although this study was conducted before the advent of modern deepfake technologies, it demonstrated early on the necessity of evaluating multiple signal types together in the verification of health-related videos.

This approach has been extended to AI-generated health deepfakes using SynthMed. Barone et al. created a multimodal corpus consisting of real and synthetic examples specific to health communication; they evaluated video, audio, and text detectors both individually and in combination. While general-purpose deepfake detectors were found to lose performance when transferred to the healthcare domain, the late-fusion approach increased macro-F1 by 0.159 and AUC by 0.131 compared to monomodal systems [19].

This result is of critical importance for the literature on healthcare deepfake detection. Detectors developed on entertainment or general social media datasets may not be sufficient when directly applied to the healthcare context. Clinical terminology, professional speech patterns, patient narratives, and content reflecting scientific evidence create healthcare-specific distributions. Therefore, domain-specific datasets and multimodal models are required.

Zhao and Zeng examined another aspect of this problem: the cost of expert labeling. In a multimodal deep active learning approach, entropy sampling, minimum confidence, and the BALD method improved F1 scores to a range of 80.73–82.60%. While the BALD approach achieved an F1 score of 0.80 using approximately 55% of the data, random sampling failed to exceed this threshold under the same conditions [68]. This demonstrates that active learning has the potential to reduce the expert workload in the creation of health misinformation datasets.

### 9.5. Source Verification, Provenance, and Watermarking

Post hoc detectors search for signs of manipulation after a piece of content has been published. In contrast, provenance and watermarking methods aim to track the production history or source of the content at an earlier stage. Zhang and Koushanfar examined watermarking methods for LLM-generated content; they noted that these methods hold potential for source tracking, ownership verification, and the identification of machine-generated content. However, resistance to attacks, the possibility of watermarks being altered afterward, and domain-specific applications remain significant limitations [69]. While synthetic medical images can be used for purposes of research, education as well as for data augmentation of clinical images, combining them with real clinical cases without proper provenance endangers the scientific quality and the safety of patients. While computational similarity metrics are not sufficient to ensure clinical validity of synthetic images, the evaluation by experts, the provenance of a dataset, watermarking as well as deepfake detection have to be considered jointly [70]. Provenance must not be confused with factual accuracy. Even a human-generated message with provenance verified to be true can be wrong, whereas clearly labeled AI-generated content may be scientifically accurate.

Table 6 summarizes the major detection approaches, reported performance metrics, and key limitations across AI-generated health misinformation and synthetic media studies.

### 9.6. Generalizability, Domain Shift, and Real-World Limitations

Strong performance on a benchmark can give a misleading impression of a detector’s reliability in routine use. A detector may achieve 98–99% accuracy on a known generator and a controlled dataset; however, it may not maintain the same performance when faced with new generator models, different clinical institutions, other languages, image compression, or social media re-encoding. The performance loss observed by SynthMed during the transfer of general-purpose detectors to the healthcare domain directly illustrates this problem [19]. Evaluation should extend beyond accuracy or AUC measured on a single dataset. Unseen-generator testing, cross-dataset validation, cross-domain evaluation, and adversarial robustness must become fundamental evaluation components in healthcare deepfake detection research. The fact that M3DSynth demonstrates a certain level of generalizability even when its training and test distributions differ [65] and that Med-GUARD maintains its advantage in cross-domain experiments [59] are positive examples in this regard. Verification against retrieved evidence has its own limitations. Scientific sources may reduce hallucinations, but incorrect, outdated, or conflicting material retrieved by the system can introduce further errors [62]. Therefore, different security mechanisms do not automatically eliminate each other’s errors.

Technical detection should support expert review, particularly when content involves high-risk health claims, manipulated clinical images, or treatment decisions. Recent syntheses of the literature on synthetic media in healthcare also demonstrate that deepfake detection is an emerging and important security capability, but verification in real clinical workflows remains limited [71].

Evaluating misleading synthetic health information online requires a number of different types of assessment. Claims in text must be verified against scientific evidence; medical images must be subjected to some form of forensic or anomaly assessment; audio and video must be assessed by detectors designed for these specific media. Assessment of multimodal online health information as well as assessment of the provenance of online health information (such as via watermarking) are also important. Current approaches and findings related to the detection of health misinformation, particularly those which are health-specific, evidence-based, and multimodal, may outperform general-purpose detectors. However, performance on a benchmark should not be confused with performance in practice, and a number of key challenges must be addressed, including the following: domain shift; detection of content generated by previously unknown generators; conflicting scientific evidence; and generalizability to real-world scenarios.

## 10. Interventions and Countermeasures Against AI-Generated Health Misinformation

Addressing health misinformation generated by AI requires intervention at several levels: after exposure of health misinformation (correction), before exposure of health misinformation (prebunking), when health users are considering sharing health misinformation (accuracy prompts), health and media literacy, and AI model safeguards. Interventions can be effective under specific conditions, with effects that vary depending on several factors, including source, message, user, and outcome. No single intervention will be effective in all contexts.

### 10.1. Debunking and Corrective Communication

Debunking seeks to correct false beliefs after a person has encountered a misleading health claim. However, the effectiveness of a correction does not depend solely on the presentation of accurate information; the source of the correction and how the message is structured also influence the outcome.

Sun and Pan compared human fact-checking, AI fact-checking, and a simple rebuttal in a factorial experiment involving 754 participants. Both human and AI fact-checking increased the intention to share corrective content compared to the simple rebuttal. Furthermore, corrections from a source different from the one that published the misinformation led to a higher intention to share, and it was shown that perceived source credibility mediated this relationship [72].

The effectiveness of AI-based corrections may also vary depending on the message format. In an experiment conducted by Tang et al. with 576 participants who initially believed the misinformation, medical experts were found to be more effective than corrections from AI-based sources or those stemming from strong social ties. However, numerical messages delivered by AI yielded stronger results, while narrative messages delivered by medical experts were more effective [73].

A study by Xie et al., consisting of three experiments, also shows that chatbots can improve the correction of health misinformation, but that the overall corrective effect of human experts remains stronger. In chatbot conditions, two-sided messages, rational arguments, explicit conclusions, and stronger fear appeals were more effective [74].

Similarly, in an experiment conducted by Xu et al. with 543 participants, two-sided corrective messages resulted in higher belief correction than one- sided messages; corrections attributed to a government health authority were found to be more effective than those from an LLM chatbot or unattributed sources [41]. These results support the view that AI-powered correction systems should be regarded as complementary tools that scale up the efforts of trusted health authorities rather than replacing them.

Personalized dialog is also an important form of intervention. In a randomized experiment conducted by Ballot et al. with 554 participants, discussing individual conspiracy beliefs related to COVID-19 with an LLM reduced belief levels compared to the control condition. Confidence in conspiracy beliefs decreased by 7.88 percentage points when the chatbot was identified as AI and by 13.76 percentage points when participants perceived their conversation partner as human [43].

### 10.2. Prebunking and Inoculation

While debunking comes into play after exposure, prebunking aims to prepare individuals against manipulative techniques before they encounter misinformation. The inoculation approach relies on explaining common misinformation techniques in advance to build cognitive resistance against future misleading messages.

In an experiment with 854 female participants, Iles et al. presented an inoculation message explaining five common characteristics of misinformation prior to exposing them to misinformation regarding breast cancer screening. Compared to the control condition, the inoculation condition reduced the negative effects of misinformation on indicators of resistance, attitudes, and intentions. In contrast, the experimental self-affirmation intervention alone did not demonstrate a protective effect [75].

Generative AI can also be used to scale up prebunking and rebuttal content. Malek et al. developed an approach using BERT, T5, and GPT-2 models to classify misinformation, identify thematic topics, and generate explanatory counter-messages targeting them. BERT achieved 98% classification accuracy, and a 44% reduction in the false-positive rate for AI-generated misinformation was reported. However, the study was conducted on offline COVID-19 datasets, and the impact of the generated interventions on actual user behavior has not been tested [60].

It is important to make a distinction between a system generating good rebuttals (persuasive or appropriate) and users actually becoming more resistant to future misinformation. Thus, assessing the performance of AI-supported prebunking systems also requires looking at user-level outcomes (beliefs, sharing, health-related decisions, etc.) in the long run and their sustainability.

### 10.3. Accuracy Nudges and Warning Labels

Accuracy-nudge interventions, which prompt users to reflect on accuracy immediately before a sharing decision, offer a low-cost and scalable approach. An experiment conducted by Xue et al. with 1393 valid participants demonstrated that negative emotional messages and fake authoritative sources increased the perceived credibility of health misinformation and the intention to share it. The accuracy-nudge label, on the other hand, encouraged verification behavior and reduced the intention to share misinformation [76].

However, not all labels serve the same epistemic function. The “AI-generated” label explains the origin of the content; it does not determine whether the content is true or false. In studies by Ji and Zhang, AI-generated debunking texts were found to be more persuasive than human-written texts in terms of logical and emotional persuasion, but were rated lower in terms of source credibility. Additionally, the “AI-written” label reduced the perceived persuasiveness of both AI- and human-written messages [42].

Therefore, the AI-origin label should be distinguished from the accuracy warning. The former provides transparency and source information; the latter encourages the user to evaluate the accuracy of the claim. Their effects may therefore differ according to whether they communicate content origin or prompt an assessment of factual accuracy.

### 10.4. Health and Media Literacy

Longer-term interventions aim to strengthen users’ ability to assess health claims independently. This requires more than factual knowledge alone. It also encompasses skills such as evaluating the quality of evidence, verifying sources, recognizing manipulative rhetoric, and understanding the limitations of digital content.

Ringle and Jensen-Doss evaluated a short, story-based critical health literacy podcast in a randomized controlled trial involving 250 U.S. parents. Compared to the control group, the intervention increased critical thinking about health claims (B = 2.56; *p* < 0.001), strengthened intentions to engage in behaviors consistent with critical thinking (B = 0.252; *p* = 0.001), and was associated with more evidence-based treatment preferences [77].

Platform-specific interventions may also be beneficial. In an educational video study developed by Alsaad and Aldossary for WhatsApp users, 483 participants showed a significant increase in their ability to identify health misinformation following the intervention. Participants demonstrated a higher level of knowledge regarding verification behaviors such as checking links, looking for “forwarded” labels, evaluating visuals, and examining facts in the content [78].

In contrast, using generative AI itself as a media literacy tool yielded unexpected results. In a 3 × 2 × 2 online experiment by Peng et al. involving 139 participants, analyses of persuasion strategies generated by ChatGPT 3.5 did not yield a significant difference in the evaluation of either accurate or inaccurate information when compared to the National Library of Medicine checklist. However, participants in the ChatGPT condition showed significantly lower accuracy in assessing the reliability of false information compared to the control group; they did not differ from the control group when evaluating true information. The authors suggest that this result can be explained by distrust of ChatGPT and psychological reactance. The small size of the ChatGPT group (*N* = 31) and the fact that participants read pre-generated, error-checked analyses rather than interacting with the tool limit the generalizability of the findings [45].

Available evidence suggests that the protective effects of media literacy vary across outcomes. Hu et al. conducted a cross-sectional study with 693 participants. Higher levels of media literacy were associated with better discrimination between reliable and unreliable information. However, at higher levels of media literacy the continued influence effect even increased after correction for several factors [79]. Thus, media literacy interventions not only need to enhance users’ confidence but also need to foster users’ awareness of uncertainty and correctability.

### 10.5. Model-Level Safety Measures

User-level interventions come into play after misinformation has been generated or published. An additional layer of defense in generative AI systems involves limiting the production of harmful health disinformation at the generation stage.

Safeguards were inconsistent across systems: Claude 2 consistently declined to generate the requested health disinformation, whereas GPT-4 via ChatGPT, PaLM 2/Gemini Pro, and Llama 2 generated such content; GPT-4 via Copilot initially refused but no longer did so at the 12-week follow-up [17]. These results demonstrate that model-level safeguards constitute a separate layer of defense distinct from user-focused interventions. However, security filters alone are not sufficient. Systems must be regularly reevaluated against evolving prompt techniques and new forms of misinformation. Selected interventions and their main findings are tabulated in Table 7.

### 10.6. The Need for Multi-Layered Interventions

The reviewed evidence points to different strengths and limitations for each intervention. Debunking may reduce false beliefs after exposure, but it does not provide automatic protection against future misinformation. Prebunking can build resilience against manipulation techniques, but it does not determine the accuracy of specific or new health claims. Accuracy nudges can encourage evaluation before sharing, but their effectiveness depends on the characteristics of the message and user behavior. Media literacy interventions can foster more general skills, but their effects vary across different outcome domains.

Variation is also evident in the wider intervention literature. A review of 50 COVID-19 misinformation studies found evidence supporting accuracy prompts, debunking, media literacy tips, warning labels, and overlays. Differences in study design, misinformation type, and measured outcomes nevertheless restricted generalization. Only 18% of the studies reviewed evaluated outcomes directly related to public health, such as vaccination intent [80].

In their synthesis of generative AI research, Saeidnia et al. describe technical, socio-technical, and governance approaches as an evolving area with limited evaluation in real-world settings. There is a particular need for further evaluation of multimodal, multilingual, and health-specific interventions [8].

These findings support combining measures that address different stages of the problem. Model safeguards can limit harmful output, platforms can provide accuracy warnings and reliable corrections, and user interventions can develop prebunking skills, critical health literacy, and AI literacy. The use of AI-based corrections in conjunction with trusted health professionals and institutions for high-risk health claims constitutes a strategy more aligned with the current evidence.

These methods have different uses in healthcare and have different implementation requirements. Verifying content provenance supports authenticity assessment but does not establish factual accuracy. Automated detection can quickly scan for issues, but is vulnerable to evasion and classification errors. Preparing people for misleading information can help them resist it, and understanding health information can help people make better decisions. However, neither of these methods can independently verify diagnostic accuracy. Whether these methods can be used in everyday healthcare depends on how well they can be integrated into existing systems, how much oversight they need, and how many resources are available. Evidence of their effectiveness in routine healthcare settings remains limited.

## 11. AI-Generated Health Misinformation and the Ethical, Legal, and Governance Dimensions of Deepfakes

Health content produced synthetically brings a host of issues to bear on privacy, consent, professional identity, liability, and the institutional trust of health organizations. Are clinician faces and voices being copied and reused? Are patient images being reused in health synthetic content? Is synthetic content being falsely attributed to health organizations? The whole lifecycle of synthetic content must be governed to remove it as it is deemed to be harmful. Who is the source of the synthetic content? What legal and ethical authority has been used to process the biometric material from which the content has been generated? How will the content be labeled? Who will be held liable for any harm caused by the synthetic content?

### 11.1. Consent, Privacy, and the Secondary Use of Synthetic Identity

Generative AI can both protect identities of patients by means of synthetic data and faces and can facilitate re-identification, biometric fraud, and the unauthorized use of personal characteristics in synthesized data. Thus, synthesis in itself does not automatically imply anonymity or ethical use.

Synthetic ophthalmological images could be used to overcome data scarcity problems in multicenter studies while at the same time protecting patient data. Even after synthetic data generation, however, there are several risks such as re-identification, inherited biases from the training data, malicious use of deepfakes for medical purposes, and unclear ownership of generated data [70]. The authors note that computational similarity alone does not demonstrate clinical validity; expert assessment, data provenance, watermarking, and prospective validation must be considered together.

Some healthcare applications use deepfake techniques for ethically legitimate purposes. Zhu et al. used face-swapping methods on clinical videos of Parkinson’s patients to conceal patient identities and demonstrated that diagnostically important body landmarks could be largely preserved while the face was altered [81]. This application shows that deepfake technology can function not only for deceptive purposes but also as a privacy-preserving data-sharing tool.

Similarly, Li et al. tested three face-swapping models on epileptic seizure videos. The altered faces were no longer recognizable, while seizure semiology was largely retained. Expert assessment favored GHOST over the other models [82].

These applications show why purpose matters in ethical assessment. Protecting a patient’s identity when sharing clinical data and inserting a physician’s face into an unauthorized treatment endorsement may involve similar techniques, but differ in consent, intent, potential harm, and deception. Therefore, governance should evaluate the context of use and the potential for rights violations rather than categorically banning the technology.

### 11.2. Synthetic Identity and the Impersonation of Healthcare Professionals

Healthcare deepfakes can make a false claim appear credible by attaching it to a professional identity; The use of a physician’s face voice or institutional identity in synthetic content allows misinformation to borrow from existing clinical authority. In such a scenario, the harm is not limited to the acceptance of the false claim; the reputation of the impersonated individual and trust in the broader healthcare system may also be compromised.

Malkoç calls this mechanism “embodied authority transfer” and describes deepfake use of real clinicians’ faces or voices as the biometric layer of AI-generated health misinformation [18]. The study identifies source identity verification, the protection of clinicians’ biometric features, and cross-platform governance as necessary measures at the system level.

Within this framework; professional identity serves as both personal information and a basis on which audiences judge health. information. The unauthorized replication of a physician’s identity thus becomes a broader problem than a classic privacy violation. The biometric rights of the person being impersonated, their professional reputation, and patients’ ability to verify the source are all simultaneously affected. Therefore, healthcare institutions’ deepfake governance must extend not only to content moderation but also to professional identity verification.

### 11.3. Responsibility, Legal Liability, and Human Oversight

Responsibility; becomes harder to assign when generative AI contributes to healthcare communication. When an erroneous AI outputt influences a patient’s decision, responsibility may be attributed to the developer; the healthcare professional using the system, the healthcare institution, the platform, or the user at varying levels; This issue is particularly pronounced in autonomous or semi-autonomous systems.

Dompmartin and Haykal identify algorithmic bias, data ownership, privacy, accountability, and clinician responsibility as key ethical and legal issues in AI-based healthcare tools. The fact that constantly updated AI systems do not conform to traditional, more static medical device regulatory models further complicates legal oversight. The authors recommend meaningful human oversight, with AI supporting clinical expertise rather than replacing it [83].

The American Geriatrics Society’s 2026 position statement applies these concerns to clinical governance. It recommends oversight throughout the GenAI lifecycle, including transparency, documentation, privacy protection, clinician involvement, and monitoring before, during, and after deployment [84]. The statement emphasizes preserving clinical judgment and relational care. Responsibility for the accuracy of AI-generated health messages extends beyond the model developer. When the message is republished by a healthcare institution or professional, institutional and professional responsibility also come into play. Therefore, accountability should be addressed as a chain-like structure encompassing each stage of production, distribution, and clinical use, rather than a linear model directed at a single actor.

### 11.4. Transparency, Disclosure, and Verification of Content Provenance

Transparency in synthetic health content concerns both its producer and the scientific evidence supporting its claims. AI labeling provides only a partial answer to the first question. Disclosing that a piece of content was generated by AI does not prove it is false; similarly, the fact that it was created by a human does not guarantee its accuracy.

Content provenance and disclosure therefore require assessment alongside, but separately from, factual accuracy. Keskinbora and Esen describe provenance records and watermarking as ways to support the scientific integrity of synthetic medical data [70]. However, the primary function of these tools is to verify the content’s production history; a separate clinical accuracy assessment must also be conducted.

Professional use of social media also requires transparency. The SAGES Ethics Committee’s framework recommends clear disclosure, identification of conflicts of interest, rigorous consent and de-identification practices for patient content, and institutional monitoring of healthcare professionals’ social media use [85]. In addition to these existing challenges, AI-enabled amplification of information raises new professional boundaries and issues of privacy.

Labeling AI-generated content with “AI-origin” is not sufficient to provide meaningful transparency. In addition to identifying the source of the content, a user needs to know whether content has been generated synthetically, whether financial or commercial interests of authors or third parties could influence the content, and whether the content is based on sufficient empirical evidence.

Implementing these measures in clinical settings, however, is very challenging from a technical as well as organizational perspective. Provenance verification requires the presence of interoperable systems capable of preserving all metadata in all environments (imaging systems, EHR systems, institutional networks). Loss of metadata during simple file conversion or transmission can render content irretrievable. However, the absence of provenance information does not necessarily mean content has been manipulated. Allowing human verification of content requires clear assignment of responsibility, sufficient clinical expertise to verify content, and time to verify content independently. Verification mechanisms thus need to be able to distinguish true positives from false positives, to be updated to newly emerging manipulation techniques, and to operate within the regular time constraints of clinical environments. Effective implementation thus not only requires resources but also integration into existing workflows and processes that can be validated in clinical settings.

### 11.5. Platform and Institutional Governance

The spread of AI-generated health misinformation involves networks of content and users across a wider information environment. The same synthetic claim can be reproduced across different accounts, platforms, and modalities. This situation renders it insufficient to reduce governance to mere decisions on individual content removal.

Malkoç’s MEDF; framework examines the temporal and systemic aspects of this problem. Repetitionn of the same false claim by many apparently independent synthetic sources can create the appearancee of an established consensus. Therefore, the author proposes structural governance includingg source identity verification cross platform coordination, and a proactive trust infrastructure in addition to interventions aimed at changing individual user behavior [18].

Professional organizations also have a monitoring role; The SAGES framework proposes institutional and Professional association mechanisms for overseeing official health messages [85]. This approach suggests that healthcare organizations may be responsible not only for correcting misinformation but also for monitoring how their professional identities are represented.

Platform governance must also account for unequal access to digital health literacy, verification tools, and regulatory support across societies. Consequently, governance models that rely solely on users to verify content may shift the burden of misinformation onto groups with lower digital capacity. A more equitable approach to health deepfakes should distribute the responsibility for verification among content creators, platforms, and health institutions before placing it on users.

### 11.6. Regulatory Gaps and the Oversight of Dynamic Systems

Many regulatory approaches assume a relatively stable product, an identifiable manufacturer, and a defined intended use. Generative AI, however, has a constantly evolving risk profile due to model updates, new prompts, and user-generated derivative content. This renders the traditional pre-market evaluation approach insufficient on its own.

Dompmartin and Haykal identify continuous AI updates as a challenge for compliance with existing health regulations [83]. The AGS position paper addresses this issue through lifecycle governance; it proposes monitoring and accountability mechanisms during and after deployment, in addition to pre-market validation [84].

Deepfake applications raise further regulatory questions. In their ethical and legal analysis of deepfake therapy, Hoek et al. describe a possible conflict between therapeutic benefit and the privacy and portrait rights of the person represented. The authors argue that the application can only be justified if it is an effective treatment option, is conducted under the supervision of a therapist, and measures are taken to protect the privacy of the person depicted [86].

This example shows why regulation of synthetic health content requires more than a distinction between real and artificial material. The same technology can protect patient privacy in one context while leading to a violation of identity or reputation in another. Regulatory assessment must therefore consider not only the type of content but also its purpose, consent, target audience, potential for harm, and context of use. Key ethical, legal, and governance considerations for generative AI and deepfakes in healthcare are summarized in Table 8.

### 11.7. Risk-Based Governance for Synthetic Health Content

The existing literature shows that synthetic health content cannot be reduced to a single ethical or regulatory category. Deepfake technology can be used to conceal a patient’s identity, as well as to imitate a real physician’s professional authority without permission. Similarly, while synthetic data can support privacy in research, it can also undermine the reliability of scientific records without proper provenance and verification. Therefore, risk must be assessed not only based on the technology used, but also on its intended purpose and outcomes.

In a risk-based governance model; at least four elements must be addressed together. Identity and consent: the use of biometrik or clinical material must be based on explicit authorization. Source and provenance: the user must be able to understand the origin of the synthetic content and whether it has a genuine connection to the person or institution it represents. Accountability requires clearly assigned responsibilities for developers, platforms, healthcare institutions, and professionals before deployment. Ongoing oversight: due to the dynamic nature of generative AI systems, evaluation must continue not only prior to market or clinical entry but throughout the duration of use [83,84].

Governance should protect legitimate clinical and research uses while restricting deceptive identities, nonconsensual biometric exploitation, and fabricated health authority. This is also where the most critical regulatory gap regarding AI-generated health misinformation emerges: while existing mechanisms largely focus on the misinformation itself, how to manage the identity and authority signals carried by synthetic content has not yet been sufficiently institutionalized [18]. Therefore, future health governance must make the protection of synthetic identity, source authenticity, and institutional trust beyond mere content accuracy an explicit regulatory objective.

Despite recent advances in research on generative AI misinformation, medical image manipulation, synthetic impersonation, and diagnostic errors, important evidence gaps persist. Most findings derive from controlled experiments or benchmark evaluations, with limited evidence concerning real-world exposure, longitudinal behavioral effects, and patient outcomes. Further clinical validation is needed to establish the generalizability of these findings across healthcare settings and to determine whether identified technical vulnerabilities translate into measurable clinical harm.

## 12. Research Gaps and the Future Research Agenda

Research on AI-generated health misinformation is growing quickly, but publication volume alone does not establish a mature evidence base. A significant portion of current studies relies on controlled experiments, the evaluation of model outputs, or specific benchmark datasets. In contrast, the causal chain linking exposure in real-world platform environments to user behavior, health decisions, and clinical outcomes has been less thoroughly examined. Recent systematic reviews also indicate that future studies should prioritize multimodal, multilingual, and health-specific validation, as well as the evaluation of interventions under real-world conditions [8].

Research priorities need to extend beyond classifying additional AI-generated content in controlled laboratory settings. The main requirement is the development of an evidence infrastructure capable of explaining under what conditions, on whom, through which platform mechanisms, and leading to what health outcomes synthetic health information operates.

### 12.1. From Benchmark Performance to Real-World Evidence

Technical studies commonly report accuracy, F1, or AUC as their main performance measures. While these metrics are necessary for algorithmic comparison, they do not, on their own, demonstrate usability in real-world social media environments. Platform compression, re-encoding, screen capture, content editing, new generative models, and diverse user languages are constantly altering the data distribution. Recent systematic reviews in the general deepfake literature also indicate that multimodal detection, cross-dataset generalization, and the balance between explainability and robustness remain unresolved challenges [87].

Health-specific detectors should be evaluated on more than random train-test splits. Testing needs to cover unfamiliar generators, different platforms and datasets, temporal changes, and post-processing. Performance should be assessed not only by technical accuracy but also by the clinical cost of false positives and the potential impact of false negatives on patient safety. Such an approach will bridge the current gap between “high benchmark accuracy” and “operational reliability.”

### 12.2. From Single-Modality to Multimodal Synthetic Healthcare Ecosystems

A significant portion of the literature treats text, images, video, or audio as separate problems. However, real-world health misinformation is increasingly multimodal in nature. A single piece of content may simultaneously use a realistic-looking video of a physician, a cloned voice, a professional explanatory text, and fabricated scientific sources. In such cases, while each modality may appear plausible on its own, the inaccuracy may become apparent only when the relationships between the modalities are evaluated.

Saeidnia et al.’s systematic review identifies multimodal and health-specific verification as key priorities for the future [8]. The general deepfake literature also reports that technical studies and the social and governance dimensions have largely developed in isolation from one another, and that there is a need for robust multimodal benchmarks [87].

Future benchmarks should therefore not consist solely of media files labeled “real/fake.” For the same example, the truthfulness of the claim, the authenticity of the source, the speaker’s identity, the scientific evidence used, cross-modal consistency, and the type of manipulation should be presented as separate annotation layers. This structure will also help methodologically distinguish between the detection of AI origin and the verification of health claims.

### 12.3. Measuring the Transition from Exposure to Actual Health Behavior

One of the most significant evidence gaps identified in this review is that the link between exposure and clinical harm has not been sufficiently examined. Many studies measure intermediate outcomes, including perceived credibility, belief in misinformation, sharing intention, and treatment preference. In contrast, actual medication use, treatment discontinuation, delays in seeking professional healthcare, or measurable clinical outcomes are investigated less frequently. Shankar et al.’s 2026 systematic review protocol proposes a synthesis of qualitative evidence on patients’ reasons for consulting LLMs, their trust or skepticism, and the influence of AI-generated information on health decisions [88]. As a protocol, it identifies questions for investigation but provides no completed synthesis from which to infer the extent of the evidence gap. Research should measure exposure, belief, reliance, decision-making, behavior, and clinical outcomes as separate stages in the proposed pathway. In particular, longitudinal cohorts, digital behavior logs, designs linked to electronic health records, and ethically feasible natural experiments could help bridge this gap. While self-reported intention is important, it should not be used as a substitute for actual health behavior.

### 12.4. Direct Investigation of Synthetic Clinicians and Artificial Authority

Synthetic clinicians are central to this review’s framework, yet few of the identified health communication studies directly test how they influence audiences. The literature primarily investigates the persuasiveness of AI-generated text or general perceptions of deepfakes. In contrast, controlled comparisons in which the same false health claim is presented by a real clinician, an unknown person, an obviously synthetic avatar, and an unauthorized clinician deepfake are quite limited.

Malkoç’s conceptualization of “embodied authority transfer” suggests that the use of real clinicians’ faces and voices may rely not only on the content but also on existing professional trust [18]. However, a significant portion of the relationships posited by this framework requires direct empirical testing.

Future experiments should systematically manipulate factors such as source identity, professional credentials, facial realism, voice cloning, disclosure, and message accuracy. Outcome variables should not be limited to the question “Do you believe this video?”; source recall, trust in the physician, trust in the healthcare system, verification behavior, sharing, and healthcare decision-making should all be examined together. In this way, research can be structured to measure the impact of synthetic authority not only on perceived trust but also on verification behavior, diagnostic decisions, professional referrals, and clinical safety.

### 12.5. Multilingualism, Cultural Context, and Health Inequalities

The evidence reviewed is unevenly distributed across regions and languages, despite the global relevance of AI-generated health misinformation. The existing evidence base on health misinformation consists largely of English-language content from high-income countries and major technology platforms. As such, it has limited ability to describe or support work aimed at the production and countering of misinformation in other languages and health systems. Omar et al. describe how low digital health literacy, poor regulatory environments, the increasing use of social media for health information seeking and the paucity of effective fact-checking in health information environments in countries such as Somalia create an environment where health misinformation generated by AI has the potential to cause harm [89]. Although this study is a research letter and therefore does not provide causal or quantitative evidence, it demonstrates why resource-constrained settings warrant separate investigation in the current empirical literature.

Therefore, future studies should not rely solely on translations of existing English-language benchmarks. Local health beliefs, trust in healthcare professionals, the use of messaging apps, language characteristics, and access to the healthcare system must be evaluated collectively. Multilingual studies should also report whether detector and fact-checking systems experience a decline in performance in languages other than high-resource ones.

### 12.6. From Cross-Sectional Studies to Longitudinal and Platform-Level Research

Single-session online experiments, commonly used in misinformation research, are suitable for measuring short-term cognitive effects; however, they cannot explain the effects of repeated exposure over time. This limitation is particularly significant because generative AI enables the same narrative to be produced repeatedly and in different forms at low cost.

In their recent synthesis of the social media and health literature, Ye and Ho emphasize that future research must shift from cross-sectional designs to longitudinal and causal approaches, assess platform-specific effects, and use objective behavioral measures in addition to self-reports [90]. Furthermore, the role of generative AI in health communication and misinformation is directly identified as a priority for future research.

When this approach is applied to health misinformation research, it is necessary to evaluate not just the impact of a single post, but the “information diet” a user encounters over time. Further research should follow content across platforms and examine both algorithmic recommendations and the cumulative effects of repeated exposure to synthetic messages.

### 12.7. Real-World Effectiveness of Interventions and Governance Mechanisms

Detection, debunking, labeling, prebunking, provenance, and watermarking address different problems, but most studies evaluate these approaches separately. Current systematic evidence shows that mitigation strategies are promising but have been evaluated unevenly, and real-world testing has been insufficient [8].

Research needs to establish where, for whom, and for how long an intervention remains effective, as well as which forms of misinformation it addresses. Furthermore, factorial studies combining platform labels, AI disclosure, expert correction, and provenance information could demonstrate whether a layered intervention architecture truly provides additional benefits.

Governance faces a comparable gap between principles and implementation. Moyo et al.’s systematic review describes fragmented work on technical detection, social effects, and regulation, with limited translation of governance principles into operational systems [87]. Future research should move beyond proposing governance principles and evaluate whether mechanisms such as source verification, provenance, and platform accountability produce measurable real-world effects.

### 12.8. Toward a Standardized Research Agenda

Inconsistent definitions and outcome measures make comparisons across studies difficult. Terms such as “AI-generated misinformation,” “deepfake,” “synthetic media,” “hallucination,” and “fabrication” are sometimes used interchangeably in some studies. Similarly, success is expressed through a variety of different outcomes, such as detection accuracy, perceived credibility, belief change, sharing intention, or clinical safety. A common reporting format should specify the generative model and version, content modality, misinformation type and verification method, exposure and source conditions, and measured user or clinical outcomes. For technical studies, cross-dataset and unseen-generator performance should also be reported; for behavioral studies, actual behavior or follow-up measurements; and for intervention studies, the sustainability of the effect (Table 9).

This agenda calls for technical and behavioral research to examine connected stages of the same proposed causal pathway. In particular, we must avoid equating production capacity with actual prevalence; perceived reliability with behavior; and technical detection success with clinical safety. The next phase in this field should focus less on demonstrating how persuasive the content generated by models can be, and more on producing direct evidence regarding what this content actually does within real-world health information ecosystems.

## 13. Limitations

- This narrative review did not use duplicate independent screening or a formal risk-of-bias assessment. Study selection may therefore have been influenced by the authors’ judgment.
- Some model versions assessed in the included studies have already been superseded as the field has developed. Reported performance estimates should therefore be interpreted as evidence of potential failure modes rather than as current error rates for specific products.
- Restricting the searches to Web of Science and Scopus may have left relevant work from other databases, languages, preprint repositories, and technical venues underrepresented.
- Disease-specific studies were selected to illustrate different diagnostic safety failures; they do not provide coverage of every clinical specialty.
- The included studies differed in design, outcome measures, model versions, and reference standards. This heterogeneity precluded meaningful quantitative pooling.

## 14. Conclusions

The reviewed literature describes risks that extend from inaccurate text to fabricated evidence and impersonated or invented professional identities. Together, these elements can give misleading health claims an appearance of authority. For RQ1, the reviewed studies show that generative AI can lower the cost of producing tailored false claims, fabricated citations and testimonials, and clinician deepfakes. For RQ2, credibility and verification depend on source authority, perceived AI competence, emotional framing, and cognitive load; persuasiveness varies with context. Evidence relevant to RQ3 is strongest for production capacity and intermediate outcomes such as trust and reliance, while direct evidence of health behavior change and clinical harm remains limited. For RQ4, detection, verification against scientific evidence, provenance, corrective communication, prebunking, literacy interventions, and governance offer complementary safeguards. Their evaluation, however, has largely relied on benchmarks and short-term experiments. Finally, the disease-specific evidence addressing RQ5 shows that high average accuracy can coexist with missed referrals, important omissions, hallucinated imaging findings, and poor calibration. In radiology, the diagnostic evidence itself may also be manipulated.

For clinical practice, generative AI systems should be validated for specific tasks against appropriate reference standards; critical error rates, referral safety, completeness, and calibration should be reported alongside overall accuracy; and externally sourced medical images should undergo provenance and authenticity assessment when clinically relevant. Clinicians should also consider asking patients whether they have used generative AI tools to obtain information about symptoms, diagnoses, or treatment, particularly because AI use may remain undisclosed during clinical encounters [31]. Such discussions should adopt a nonjudgmental approach that encourages disclosure without undermining patient trust. Patient-provided AI-generated information should be assessed against established clinical evidence, with source authenticity verified when relevant. Although this approach may support shared decision-making, its effectiveness in routine clinical practice requires further evaluation. The central question is therefore not whether generative AI can produce health information, but under which conditions synthetic claims, evidence, and identities alter diagnostic and therapeutic decisions.

## Figures and Tables

**Table 1:** Key distinctions in the evaluation of AI-generated health content.

Evaluation Dimension	Guiding Question
Content accuracy	Is the health claim consistent with the best available evidence?
Intent to deceive	Is there evidence that the content was produced or disseminated with the intent to mislead?
Mode of production	How was the content generated, modified, or manipulated?
Identity authenticity	Does the purported speaker or source actually exist, and is the content genuinely attributable to that individual or entity?
Authority and experience	Are the presented expertise, endorsement, or patient experience authentic?

**Table 2:** Forms of AI-generated health misinformation and the limitations of the current evidence.

Form	Misleading Use	Evidence and Study Design	Interpretive Limitation
Text and bibliographic sources	Addition of fabricated scientific support to a health claim.	Analysis of fabricated citations and bibliographic errors [20].	Rates obtained from specific models and prompts cannot be generalized to all generative AI systems.
Synthetic images	Presentation of a generated image as evidence of a real event or clinical finding.	MM-Health dataset for text–image generation and evaluation [6].	Dataset-level results do not measure whether users believe, verify, or share the generated content.
Deepfake videos	False attribution of a health-related statement to a medical expert.	Online experiment in the context of cancer prevention [21].	Findings depend on the topic and experimental conditions; actual clinical harm was not measured.
Cloned voice	Creation of a false impression regarding speaker identity or message provenance.	Randomized study examining voice cloning in patient education [22].	Findings from a legitimate educational application do not establish the effects of deceptive voice cloning.
Physician identity	Transfer of professional credibility to a health claim through impersonation.	News and commentary sources [5,23] and a conceptual framework [18].	These sources document the phenomenon but do not permit estimation of prevalence or causal effects.
Patient testimonial	Presentation of a fabricated experience as an authentic treatment outcome.	Generation of fabricated patient and clinician testimonials using LLMs [17].	Feasibility of generation has been demonstrated, but effects on treatment preferences or clinical decisions have not been measured.
Multimodal content	Integration of text, images, audio, or video into a unified misleading health narrative.	MM-Health and SynthMed [6,19].	Technical detection or generation performance cannot be equated with effectiveness under real-world platform conditions or with behavioral impact.

**Table 3:** Documented synthetic health content and forms of deception on social media.

Platform and Report Year	Case and Health Topic	Content Presentation and Manipulation	Classification	Documented Finding and Source
Facebook and Instagram, 2024	Health-product promotions using the identities of well-known physicians, including Hilary Jones, Michael Mosley, and Rangan Chatterjee; claims related to hypertension and diabetes treatment.	Videos created to give the impression that the physicians endorsed or promoted the products.	Deepfake; commercial disinformation based on fabricated expert endorsement.	A *BMJ* investigation documented the impersonated physicians and the platforms on which the videos appeared. It did not measure prevalence or clinical impact [23].
Facebook, Instagram, and Messenger, 2024	“Miracle cure” and supplement advertisements targeting various diseases.	AI-generated images, audio, and videos; impersonation of physicians and celebrities; redirection to commercial websites.	Multimodal synthetic media; deepfake-assisted commercial disinformation.	Bitdefender Labs reported more than 1000 distinct deepfake videos in campaigns examined between March and May 2024. This figure refers only to the investigated campaigns [24].
TikTok, 2025	David Taylor-Robinson portrayed as recommending supplements for menopausal symptoms.	Manipulation of authentic conference footage and false attribution of a product recommendation to the individual.	Deepfake; fabricated expert endorsement.	Full Fact examined the original footage and the statement of the impersonated individual. One video received more than 365,000 views before removal. The investigation did not independently assess the health claims made for the products [25].
TikTok, 2026	Video portraying Garth Davis as claiming that sleep problems and intense food cravings indicate excessive sugar in the body.	Video presented using the identity of a real physician, with speech showing indicators consistent with AI generation.	Physician impersonation; misleading health inference.	Science Feedback noted that the reported symptoms are nonspecific and do not support the stated inference. The AI-detection result was treated as supporting evidence rather than definitive technical verification [26].
YouTube, 2026	Health recommendations attributed to nutrition expert Serge Hercberg on the “Santé Sage” channel.	AI-based imitation of the real expert’s appearance, voice, and identity.	Deepfake; false source attribution.	Science Feedback documented the unauthorized use of Hercberg’s identity. The verification primarily concerned speaker identity rather than the medical accuracy of each recommendation [27].
YouTube, 2026	Claim on the “Dre Ana Laura” channel that baking soda improves erectile function.	Unsupported health advice delivered by an apparently synthetic presenter introduced as a physician.	Synthetic media; health misinformation; unverifiable professional identity.	Science Feedback reported finding no research supporting the claim and was unable to verify a physician identity corresponding to the presented name. Failure to identify the person in available records does not, by itself, establish that the individual does not exist [27].

**Table 4:** Selected evidence on diagnostic safety and clinical impact in disease-specific generative AI applications.

Study	Clinical Area	Design/Data	Main Finding	Implication for Diagnostic Safety
Kollayan and Cebeci [28]	Oral oncology	20 scenarios; 7-day repeated-prompt evaluation; 280 responses	Referral safety was 90.0% for Gemini and 92.1% for Grok; Gemini failed to recommend professional evaluation in two high-risk malignancy scenarios.	High average performance does not exclude the risk of critical under-referral.
Ismayilov et al. [31]	Oncology	400 patients and caregivers	Twenty-four percent used AI for cancer-related information; 69% of users did not disclose this use to their physician.	Undisclosed AI use may allow AI-generated information to influence decisions outside the clinical encounter.
Fereydooni et al. [32]	Prostate cancer pathology	44 pathology reports; 24 independent test reports	A structured GPT-4-turbo approach reduced report complexity while maintaining accuracy under test conditions.	Narrow, controlled, and validated tasks may represent safer use cases.
Wu et al. [33]	Chronic cough	6 chatbots; 25 patient questions	Pooled accuracy was 80.39%, but clinically important details were frequently omitted.	Accuracy should be evaluated together with completeness and guideline concordance.
Iritas [34]	Dermatology	3 models; 12 androgenetic alopecia questions	ChatGPT and Gemini generally performed well, whereas DeepSeek showed difficulties particularly in distinguishing androgenetic from cicatricial alopecia.	Model performance may vary according to the complexity of the clinical distinction.
Aydin et al. [35]	Adolescent scoliosis	Prospective diagnostic accuracy study; *N* = 97	ChatGPT showed 0% specificity; Claude produced 78.3% false positives; substantial Cobb angle errors were also observed.	General-purpose multimodal LLMs should not be used as standalone screening tools without task-specific validation.
Huppertz et al. [36]	Radiology	206 radiographs, CT, MRI, and angiography studies	First-diagnosis accuracy ranged from 8.3% to 29.1%; 258 fabricated imaging findings were reported across 412 responses.	The ability to process medical images does not imply reliable clinical interpretation.
Brin et al. [37]	Emergency radiology	230 real clinical images	Pathology recognition was 35.2%, with marked performance differences across imaging tasks.	Modality recognition, anatomic localization, and pathology detection should be evaluated as distinct capabilities.
Shin et al. [29]	Patient-facing radiology communication	2000 questions; 12,000 LLM responses	Terminology accuracy was 98.1%, whereas performance for diagnostic-confidence questions was 82.3%; many critical errors were delivered with high confidence.	Calibration constitutes a distinct dimension of clinical safety beyond accuracy alone.
Tordjman et al. [30]	Synthetic radiography	17 radiologists; authentic and AI-generated radiographs	Human discrimination accuracy was 70–75%; no LLM detected all synthetic images.	Diagnostic safety requires verification of image authenticity in addition to interpretation accuracy.

**Table 5:** Key empirical mechanisms underlying the persuasiveness of AI-generated health information and misinformation.

Study	Design/Sample	Mechanism	Main Outcome	Main Finding
Gu et al., 2026 [38]	Scenario experiment; *N* = 594	Automation bias; perceived diagnostic capability	Misinformation discernment intention	Higher perceived AI diagnostic capability reduced intention to scrutinize misinformation.
Reinhardt et al., 2026 [39]	Cross-national survey; *N* = 1990	AI literacy; performance expectancy; trust	Intention to use AI health information	AI literacy and perceived utility were associated with higher trust; trust was associated with use intention.
Sun et al., 2026 [40]	Survey *N* = 142 + laboratory study *N* = 40	Source label; transparency; automation	Trust	LLM-generated content received greater trust, but human labels increased trust relative to AI labels.
Xie & Li, 2026 [4]	2 × 2 × 3 experiment; *N* = 120	Authority cues; emotion; communication channel	Detection accuracy	Authoritative sources and emotional framing reduced detection of AI-generated health fake news.
Xu et al., 2026 [41]	2 × 3 experiment; *N* = 543	Source credibility; message sidedness	Belief endorsement	Government source and two-sided messages increased correction effectiveness through distinct credibility/persuasion pathways.
Ji & Zhang, 2026 [42]	Three complementary studies	Pathos, logos, ethos; AI labeling	Persuasiveness	AI texts performed better on emotion and logic but worse on source credibility.
Ballot et al., 2026 [43]	Randomized online experiment; *N* = 554	Source attribution; perceived neutrality	Conspiracy-belief revision	LLM debate reduced conspiracy beliefs; effect was stronger when the source was believed to be human.
Alhur & Alnasser, 2026 [44]	Cross-sectional survey; *N* = 487	eHealth literacy; cognitive load; self-efficacy	Verification/following intentions	Cognitive load was associated with less verification, whereas eHealth literacy and self-efficacy were associated with greater verification intention.
Peng et al., 2024 [45]	Online factorial experiment	Media literacy; persuasive-strategy awareness	Misinformation discernment	ChatGPT-generated persuasion analyses did not differ significantly from the NLM checklist but yielded lower misinformation discernment accuracy than the control condition.
Omar et al., 2026 [46]	20 LLMs; >3.4 million prompts	Authority framing; rhetorical cues	LLM susceptibility	Authoritative clinical framing increased susceptibility to fabricated medical information.

**Table 6:** Major methods, performance metrics, and limitations used in detecting AI-generated health misinformation and synthetic media.

Study	Modality/Context	Method	Key Performance	Main Contribution	Main Limitation
Pecher et al. [56]	Textual medical misinformation	Claim-based information retrieval + machine learning + expert validation	–	Introduced an explainable detection approach grounded in verified claims.	Dependent on a pool of expert-validated claims.
Hou et al. [67]	250 prostate cancer YouTube videos	Linguistic, acoustic, and engagement features	Accuracy 74%; precision 76.5%; recall 73.2%	Demonstrated the feasibility of multimodal detection in health-related videos.	Restricted to a single disease domain and a limited sample.
Abbasi et al. [63]	Synthetic skin lesions	Modified VGG16	Accuracy 99.82%	Achieved strong real-versus-synthetic discrimination under controlled conditions.	Limited to specific generators and data distributions.
Zingarini et al. [65]	Manipulated 3D lung CT images	Forensic detection and localization	8577 manipulated samples	Detected and localized nodule insertion and removal manipulations.	Requires external validation in real clinical settings.
Barone et al., CER [57]	Text, video, and web content	Claim detection + PubMed retrieval + veracity assessment	Improved F1 across benchmarks	Enabled multisource biomedical fact-checking.	Dependent on retrieval coverage.
Barone et al. [58]	Biomedical text	Evidence retrieval + LLM reasoning	State-of-the-art performance reported	Integrated evidence retrieval with reasoning.	Transfer from benchmarks to real-world settings remains uncertain.
Zhao and Zeng [68]	Multimodal social media content	Deep active learning	F1 80.73–82.60%	Achieved strong detection performance with reduced labeling requirements.	Requires expert annotation during initial training.
Barone et al., SynthMed [19]	Text + audio + video	Monomodal detectors + late fusion	Macro-F1 +0.159; AUC +0.131	Demonstrated an advantage of multimodal fusion under health-domain shift.	Limited availability of health-specific benchmarks.
Cao et al., Med-GUARD [59]	Medical claims + supporting evidence	Multi-model adversarial reasoning	+3.2–4.5 F1; hallucinations reduced by 66–70%	Enabled explainable and cross-domain verification.	Architectural and computational complexity.
Upadhyay and Viviani [61]	Consumer health information retrieval	RAG + factual-accuracy ranking	Improved over baselines	Jointly considered relevance and factual accuracy.	Dependent on the currency and quality of the underlying knowledge base.
Tariq et al. [64]	Synthetic skin cancer images	DenseNet169 transfer learning	Test accuracy 98.50%	Distinguished multiple generator types.	Broader external validation across unseen generators is required.
Grabovski et al. [66]	CT/MRI medical deepfakes	Unsupervised diffusion-based anomaly detection	AUC 0.90/0.96	Reported strong results toward generator-independent detection.	Limited to specific insertion/removal scenarios.
Javadi et al. [62]	Medical RAG	Contradiction-aware evaluation	–	Showed that contradictory or outdated evidence can reduce RAG reliability.	Evaluated within specific retrieval and medical-document scenarios.
Zhang and Koushanfar [69]	LLM-generated content	Watermarking	–	Supported source attribution and provenance tracking.	Vulnerable to adversarial removal and uncertain robustness.

**Table 7:** Empirical characteristics and key findings of selected interventions against health misinformation.

Study	Intervention	Design/Sample	Primary Outcome	Main Finding Reported in the Source
Sun and Pan [72]	Human fact-checking, AI fact-checking, and simple rebuttal	Factorial experiment; *N* = 754	Intention to share corrective content	Human and AI fact-checking generated greater sharing intentions than simple rebuttal; corrections from a different source were more effective.
Tang et al. [73]	Corrections from AI, medical experts, and strong social ties	3 × 2 experiment; *N* = 576	Correction effectiveness, credibility, and sharing intention	Expert corrections were generally more effective, while the effect of message format varied according to the source.
Xie et al. [74]	Chatbot-based debunking	Three experiments	Misinformation correction	Chatbots improved correction outcomes, although human experts remained more effective. Two-sided, rational, and explicitly concluded messages performed better.
Xu et al. [41]	Corrective messaging varying source and message sidedness	2 × 3 experiment; *N* = 543	Acceptance of the corrected health claim	Two-sided messages and corrections attributed to governmental health authorities produced greater acceptance.
Ballot et al. [43]	Personalized LLM-based debate	Randomized experiment; *N* = 554	COVID-19 conspiracy belief	Confidence in conspiracy beliefs decreased by 7.88 percentage points when the chatbot was perceived as AI and by 13.76 percentage points when it was perceived as human.
Iles et al. [75]	Inoculation message	Randomized experiment; *N* = 854	Resistance, attitudes, and intentions	The inoculation message reduced the adverse effects of breast cancer screening misinformation.
Xue et al. [76]	Accuracy-nudge label	Online factorial experiment; *N* = 1393	Misinformation-sharing intention and verification behavior	Accuracy nudges increased verification behavior and reduced intention to share misinformation.
Ringle and Jensen-Doss [77]	Critical health literacy podcast	Two-arm RCT; *N* = 250	Critical thinking, behavioral intention, and treatment preference	The intervention produced significant improvements across all three outcome domains.
Alsaad and Aldossary [78]	Educational video	Single-arm pre–post study; *N* = 483 completers	Knowledge of identifying health misinformation on WhatsApp	Knowledge of misinformation recognition increased significantly after the intervention.
Peng et al. [45]	ChatGPT-based media literacy tool	3 × 2 × 2 online experiment	Discernment of accurate and inaccurate health information	ChatGPT performed similarly to the NLM checklist but showed lower performance than the control condition for misinformation discernment.
Hu et al. [79]	Protective role of media literacy	Cross-sectional online study; *N* = 693	Credibility discernment, sharing discernment, and continued influence	Higher media literacy was associated with better credibility discernment but also with stronger continued influence effects.
Menz et al. [17]	LLM safeguards	Repeated cross-sectional model analysis	Safeguards against generation of health disinformation	Safeguards were inconsistent across systems; several publicly available LLMs generated health disinformation, and some reported vulnerabilities remained unresolved at follow-up.

**Table 8:** Selected ethical, legal, and governance resources regarding the use of generative AI and deepfakes in healthcare.

Study	Evidence Type	Health Context	Main Issue Addressed	Proposed Governance/Safety Approach
Keskinbora and Esen [70]	Narrative review	Synthetic ophthalmic data	Re-identification, bias, deepfake images, data ownership, and legal liability.	Expert clinical evaluation, provenance, watermarking, deepfake detection, and prospective validation.
Zhu et al. [81]	Empirical technical study	Parkinson’s disease clinical videos	Patient privacy in data sharing.	Face-swapping-based de-identification while preserving diagnostically relevant information.
Li et al. [82]	Empirical technical/clinical study	Epileptic seizure videos	Preserving seizure semiology while concealing patient identity.	AI-based face swapping for privacy-preserving video sharing.
Malkoç [18]	Conceptual viewpoint	AI-generated health misinformation	Impersonation of clinicians’ faces and voices, epistemic trust, and systemic effects.	Source identity verification, biometric protection, and cross-platform governance.
Dompmartin and Haykal [83]	Ethical–legal perspective	AI-based health tools	Accountability, liability, privacy, informed consent, and regulatory adaptation.	Human oversight, interdisciplinary governance, and adaptive regulation.
Bharija et al. [84]	Professional position statement	Generative AI in older adults	Misinformation, bias, privacy, and automation over-reliance.	Transparency, clinician-in-the-loop oversight, and lifecycle monitoring.
Fong et al. [85]	Ethics committee white paper	Surgeons’ use of social media	Professional identity, privacy, consent, disclosure, and misinformation.	Institutional monitoring, explicit disclosure, and stringent consent and de-identification standards.
Hoek et al. [86]	Ethical and legal analysis	Deepfake therapy	Patient benefit, reality blurring, overattachment, privacy, and portrait rights.	Therapist oversight and safeguards protecting the privacy of the depicted individual.

**Table 9:** Priority future research areas for studies on AI-generated health misinformation.

Priority Area	Current Key Gap	Recommended Research Approach	Priority Outcome Measures
Real-world generalizability	The extent to which controlled benchmark performance transfers to real-world platform environments remains uncertain.	Cross-platform, cross-dataset, unseen-generator, and temporal validation.	F1/AUC together with false-positive burden, calibration, and operational error.
Multimodal synthetic content	Text, images, audio, and video are generally evaluated separately.	Health-specific benchmarks combining the same misleading narrative across multiple modalities.	Claim truthfulness, source authenticity, identity consistency, and multimodal coherence.
Health behavior and clinical harm	Belief and intention measures are more common than assessments of actual behavior and clinical outcomes.	Longitudinal cohorts, digital behavioral records, and linkage with clinical data.	Treatment adherence, healthcare utilization, delays in care, self-medication, and clinical outcomes.
Synthetic clinicians and authority	The effects of physician face and voice impersonation have been directly tested only to a limited extent.	Factorial experiments manipulating source identity × deepfake status × disclosure × message accuracy.	Trust, verification, sharing, decision-making, and institutional trust.
Patient experiences	The meaning of LLM use in health contexts and its role in patient decision-making remain insufficiently synthesized.	Qualitative and mixed-methods studies, including repeated-use diary designs.	Motivation, reliance, skepticism, decision pathways, and clinician–patient relationships.
Multilingualism and equity	Evidence is dominated by English-language studies and high-income settings.	Multinational, culturally adapted studies including low-resource languages.	Language-specific accuracy, trust, access, and intervention effectiveness.
Longitudinal platform effects	Single-exposure study designs predominate.	Panel studies, digital trace data, and platform-specific natural experiments.	Repeated exposure, behavioral change, and erosion of trust.
Intervention effectiveness	Most strategies are evaluated in isolation and over short follow-up periods.	Multilevel factorial designs and pragmatic trials.	Actual sharing behavior, persistence of correction effects, and downstream behavior.
Provenance and governance	The real-world impact of technical standards has received limited evaluation.	Platform deployment studies and policy evaluations.	Adoption, circumvention, user comprehension, and harm reduction.
Standardization	Definitions, annotation procedures, and outcome measures remain heterogeneous.	Common reporting guidelines and standardized benchmark protocols.	Comparability, reproducibility, and external validity.

## Data Availability

No new data were created or analyzed in this study. Data sharing is not applicable to this article.
